# ADORA2A promotes proliferation and inhibits apoptosis through PI3K/AKT pathway activation in colorectal carcinoma

**DOI:** 10.1038/s41598-023-46521-1

**Published:** 2023-11-09

**Authors:** Longyan Ran, Xiao Mou, Zhenglin Peng, Xiaochen Li, Meirong Li, Duo Xu, Zixi Yang, Xingwang Sun, Tao Yin

**Affiliations:** 1 College of Basic Medicine, Southwest Medical University, Luzhou, Sichuan China; 2 College of Clinical Medicine, Southwest Medical University, No.25 Taiping Street, Jiangyang District, Luzhou City, Sichuan China; 3 Department of Pathology, Affiliated Hospital of Southwest Medical University, Luzhou, Sichuan China; 4 Present Address: Luzhou Key Laboratory of Precision Pathology Diagnosis for Serious Diseases, Luzhou, Sichuan China

**Keywords:** Cancer genetics, Gastrointestinal cancer, Oncogenes, Tumour biomarkers

## Abstract

The third most often diagnosed disease globally and the second most prevalent cause of cancer-related death is colorectal cancer (CRC). Numerous human malignancies have been identified to have high expression of ADORA2A. However, it is still ambiguous about its function in CRC. RNA-seq with stable transfected SETDB1 knockdown cells was used to identify differentially expressed genes. Further, knockdown of ADORA2A in CRC cell lines SW620 and HCT116 was performed with siRNA and over expression of ADORA2A in SW480 cells was conducted with plasmids. CCK8, colony formation, wound healing, and transwell assay were used to detect the effects of cell proliferation, migration, and invasion after knockdown and over expression of ADORA2A. Also, apoptosis was analyzed by flow cytometry, apoptosis-related proteins and key PI3K/AKT pathway proteins were detected using Western blotting. ADORA2A was identified after RNA-seq analysis and played an important role in CRC prognosis. ADORA2A was relatively high in SW620 and HCT116 cell lines compared to SW480 cell lines. ADORA2A knockdown in SW620 and HCT116 inhibited cell proliferation, migration, and invasion, while ADORA2A overexpression had the opposite effect. In addition, ADORA2A also impacted the expression of apoptosis-related proteins, including Bcl-2, Bax, Cleaved caspase-3 and Cleaved caspase-9, and reduced apoptosis. Furthermore, this process may include the PI3K/AKT signaling pathway. ADORA2A promotes CRC progression and inhibits apoptosis by the PI3K/AKT signaling pathway. It may contribute to the management and treatment of CRC.

## Introduction

In 2020, there were 1.9 million new cases of colorectal cancer throughout the world, making it the second most lethal disease^1^. Radiation, immunotherapy, palliative chemotherapy, targeted therapy, major surgery, and local ablative therapies for metastases are some of the therapeutic alternatives for CRC that have emerged recently^2,3^. However, different clinical and pathological characteristics of CRC might contribute to a variety of prognoses and likely account, at least in part, for therapy resistance^4,5^. Therefore, it is crucial to develop new treatment plans and find new molecular markers for CRC prognosis.

ADORA2A encodes the G protein-coupled adenosine receptor known as Adenosine Receptor Subtype A2a (ADORA2A). ADORA2A contact with Adenosine limits anti-tumor immunity by suppressing multiple immune subsets including T cells^6^. ADORA2A and DRD1/DRD2 increase the permeability of the blood–brain barrier by inhibiting adenylyl cyclase activity, change the level of dopamine in the brain, and finally cause tremor in Parkinson’s disease^7^. Studies have shown that ADORA2A is highly expressed in Alzheimer’s disease, and ADORA2A is an independent risk factor for Alzheimer’s disease^8^. ADORA2A gene polymorphism may affect the response to methotrexate therapy and may serve as a potential biomarker for methotrexate treatment in rheumatoid arthritis^9^. In addition, evidence has shown in recent years that ADORA2A expression is intimately linked to the development and spread of tumors. The World Health Organization (WHO) grade III gliomas in particular showed high levels of ADORA2A expression^10^. Compared to non-tumor gastric tissues and cell lines, ADORA2A expression was higher in gastric carcinoma (GC) tissues and cell lines. In addition, ADO interacts with ADORA2A to increase GC cell stemness and ADORA2A is a possible target for enhancing GC radiosensitivity^11^. ADORA2A is highly expressed in a low-risk group of pancreatic cancer, which provides a new way for immunotherapy of pancreatic cancer^12^. ADORA2A blockage immunotherapy for renal cell carcinoma that is resistant to treatment^13^. However, the function of ADORA2A in CRC remains unidentified.

In this study, we identified ADORA2A enriched in CRC progression-related pathways. Higher ADORA2A expression in CRC patients predicted poor survival. And we further explored ADORA2A expression in colorectal cancer cell lines. We found that ADORA2A knockdown decreased cell proliferation, migration, and invasion, and promoted apoptosis. Overexpression of ADORA2A had the opposite results. And the PI3K/AKT signaling pathway may be involved in the process. Conclusion ADORA2A may be a therapeutic and prognostic target of CRC.

## Results

### ADORA2A plays a vital role in CRC

**Figure 1:**
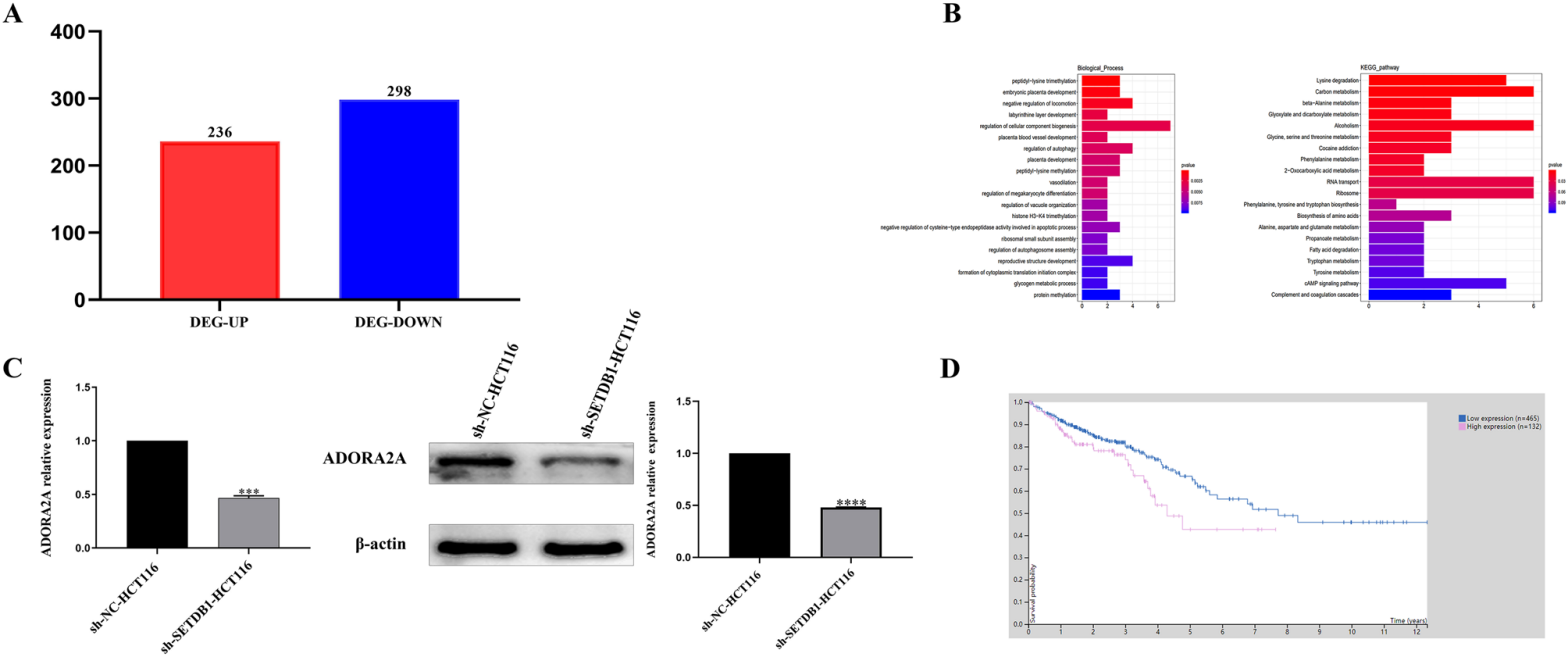
ADORA2A acts as an important progression factor in CRC. (**A**) DEGs of Stable transfected SETDB1 knockdown cells (DEG-UP: up-regulated genes with Stable transfected SETDB1 knockdown cells, DEG-DOWN: down-regulated genes with Stable transfected SETDB1 knockdown cells). (**B**) Biological process of GO and KEGG analysis with DEGs. (**C**) mRNA and protein expression of ADORA2A in colorectal cancer cells with stable knockdown of SETDB1. (**D**) Survival curve gained from HPA of CRC (low expression (n = 465); high expression (n = 132); p = 0.033).

Stable transfected SETDB1 knockdown cells were used to examine the changes through next-generation sequencing. A total of 534 differentially expressed genes (DEGs, 236 up-regulated and 298 down-regulated) were identified between sh-SETDB1-HCT116 and sh-NC-HCT116 cells (Fig. 1A). Gene function analysis with GO and KEGG revealed that ADORA2A and KMT2B were enriched in various processes related to carcinogenesis. As KMT2B was well reported in CRC, this study was mainly focused on ADORA2A^14^. Plus, ADORA2A was relatively enriched in the biological functions of the cAMP signaling pathway and negatively regulated cysteine-type endopeptidase activity involved in apoptotic processes (Fig. 1B). Subsequently, we verified ADORA2A expression in colorectal cancer cell lines with stable SETDB1 knockdown by qRT-PCR and western blotting, respectively. The results showed that the mRNA and protein expression of ADORA2A was significantly decreased (Fig. 1C). We analyzed the prognosis of ADORA2A in CRC using data from the HPA database. Results showed that high ADORA2A expression was associated with a low overall survival rate in CRC (P = 0.033, Fig. 1D). Thus, ADORA2A may be a potential prognostic factor in CRC.

### Knockdown of ADORA2A inhibits proliferation, migration and invasion of CRC cells

**Figure 2:**
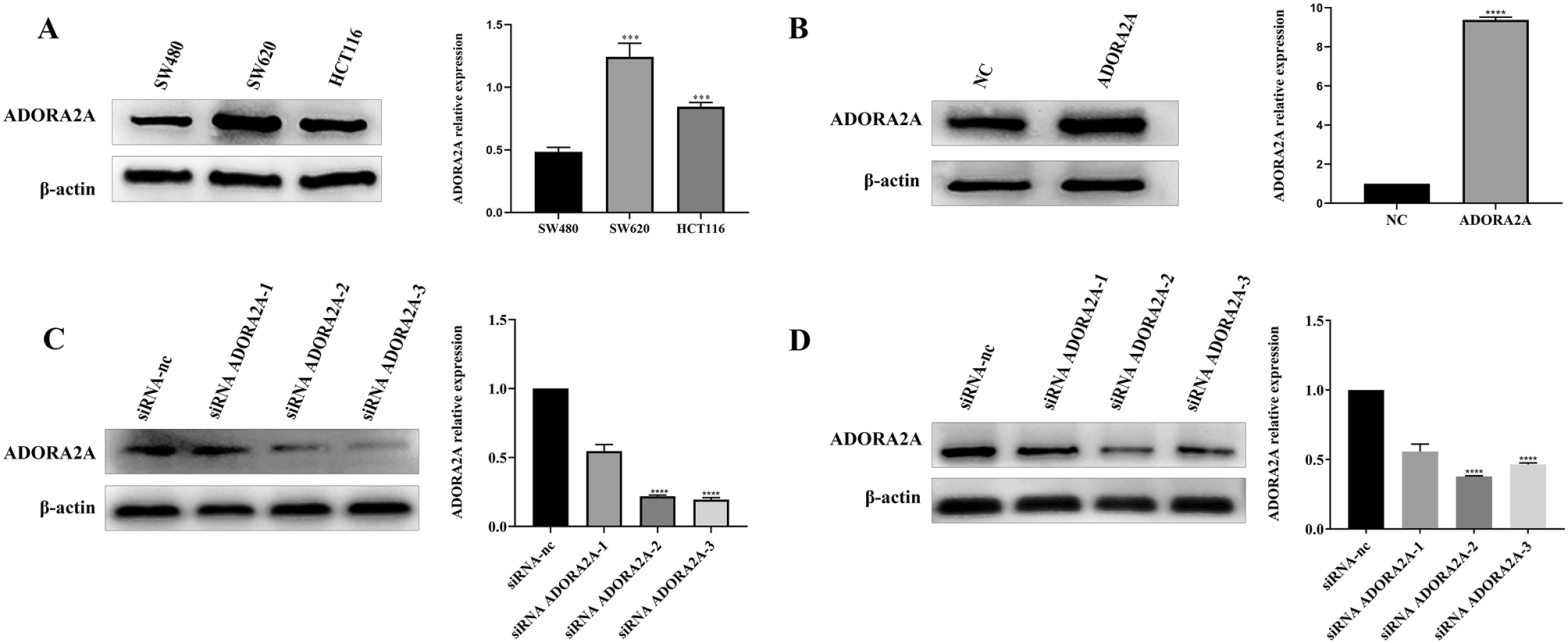
The expression of ADORA2A in CRC cells. (**A**) The expression of ADORA2A in SW620, HCT116, SW480 CRC cell lines. (**B**) Transfection efficiency validation of ADORA2A overexpression in SW480. (**C**) Validation of ADORA2A knockdown by transfection effectiveness in SW620. (**D**) Validation of ADORA2A knockdown by transfection effectiveness in HCT116.

**Figure 3:**
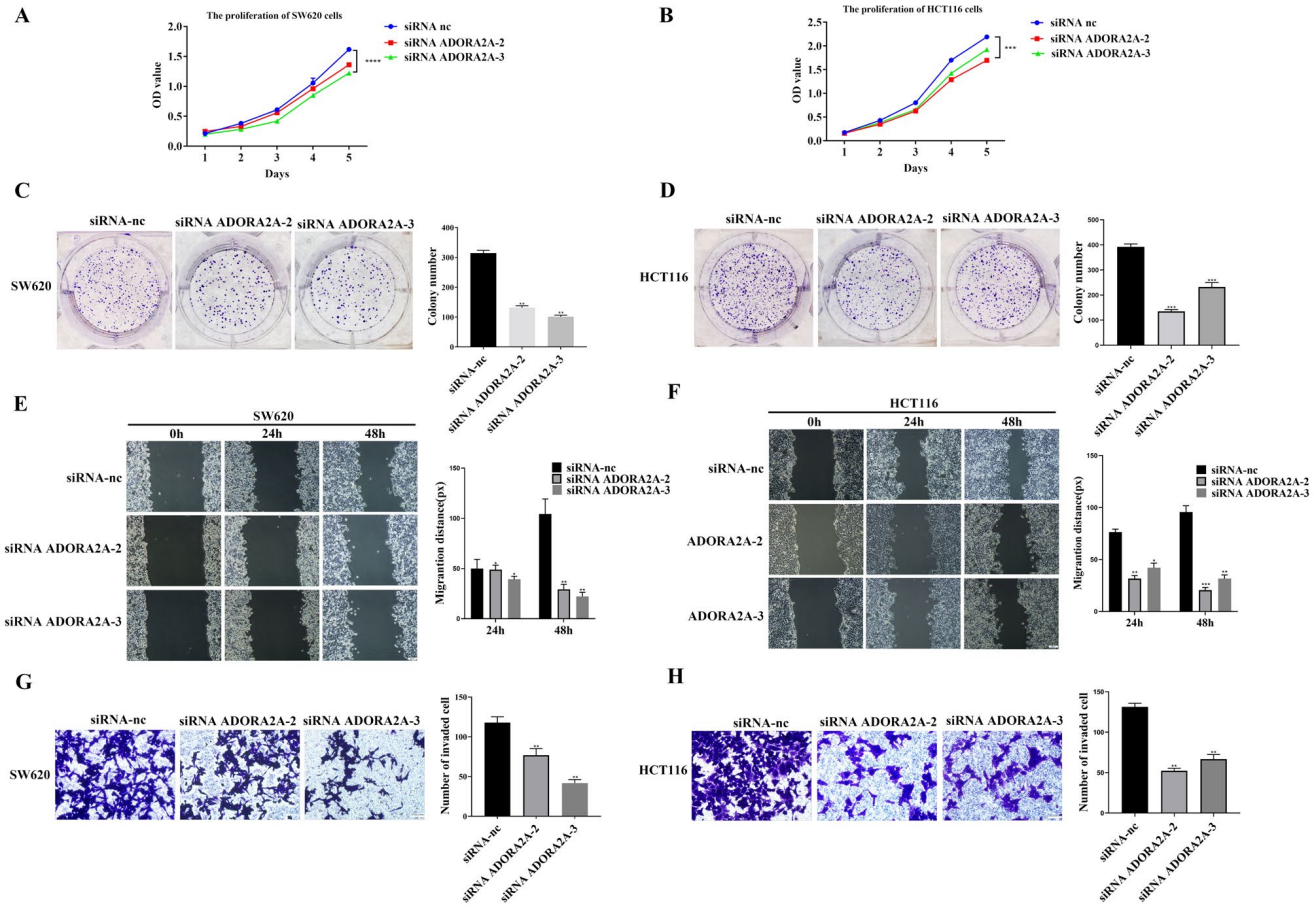
Knockdown of ADORA2A inhibits proliferation, migration, and invasion of CRC cells. (**A**–**B**) CCK8 assay of ADORA2A transfected with siRNA ADORA2A-2 and siRNA ADORA2A-3 in SW620 and HCT116. (**C**–**D**) Colony formation assay of ADORA2A transfected with siRNA ADORA2A-2 and siRNA ADORA2A-3 in SW620 and HCT116. (**E**–**F**) ADORA2A wound healing assay transfected with siRNA ADORA2A-2 and siRNA ADORA2A-3 in SW620 and HCT116. (**G**-**H**) Transwell assay of ADORA2A transfected with siRNA ADORA2A-2 and siRNA ADORA2A-3 in SW620 and HCT116. Bars:200 μm (**E**–**F**); 50 μm (**G**–**H**).

To understand the function of ADORA2A in CRC, we detected ADORA2A expression in CRC cell lines SW620, HCT116, SW480 by qRT-PCR and Western blot. ADORA2A was highly expressed in SW620 and HCT116 cells, and ADORA2A was relatively low in SW480 (Fig. 2A). We knock down ADORA2A expression in SW620 and HCT116 cells with siRNA. Compared to NC, transfection with siRNA ADORA2A-2 and siRNA ADORA2A-3 significantly reduced ADORA2A expression (Fig. 2C–D). CRC cell proliferation was dramatically reduced due to ADORA2A knockdown using CCK8 assay and tablet cloning (Fig. 3A–D). Furthermore, wound healing and transwell migration showed reduced migration and invasion (Fig. 3E–H). These findings showed that ADORA2A knockdown prevents CRC cells from proliferating, migrating, and invading.

### Over-expression of ADORA2A induces the malignant phenotypes of SW480 cells

**Figure 4:**
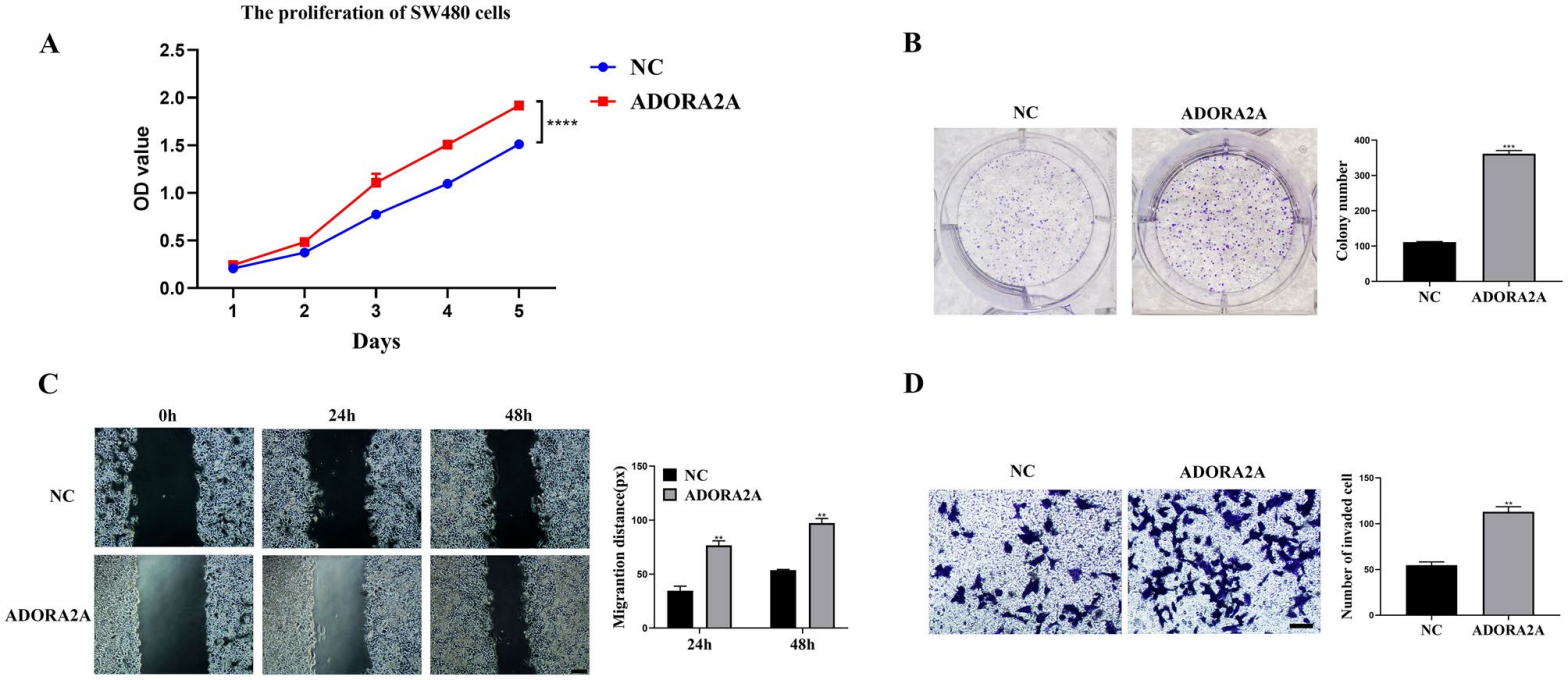
Overexpression of ADORA2A promotes malignant phenotypes in SW480 cells. (**A**–**B**) CCK8 assay and colony formation assay of SW480 cells transfected with ADORA2A overexpression plasmid. (**C**) Wound healing assay of SW480 cells transfected with ADORA2A overexpression plasmid. (**D**) Transwell assay of SW480 cells transfected with ADORA2A overexpression plasmid. Bars:200 μm (**C**); 50 μm (**D**).

Our earlier findings suggested that ADORA2A may function as an oncogene in CRC cells, and inhibiting its expression can prevent CRC cells from developing a malignant phenotype. SW480 was used for detecting biological behavior after ADORA2A overexpression. The results showed that ADORA2A expression was significantly increased after transfection with an overexpressed plasmid (Fig. 2B). ADORA2A overexpression accelerated SW480 cell proliferation, migration, and invasion (Fig. 4A–D). These findings suggested that ADORA2A overexpression is linked to CRC malignancy.

### ADORA2A inhibits apoptosis of CRC cells

**Figure 5:**
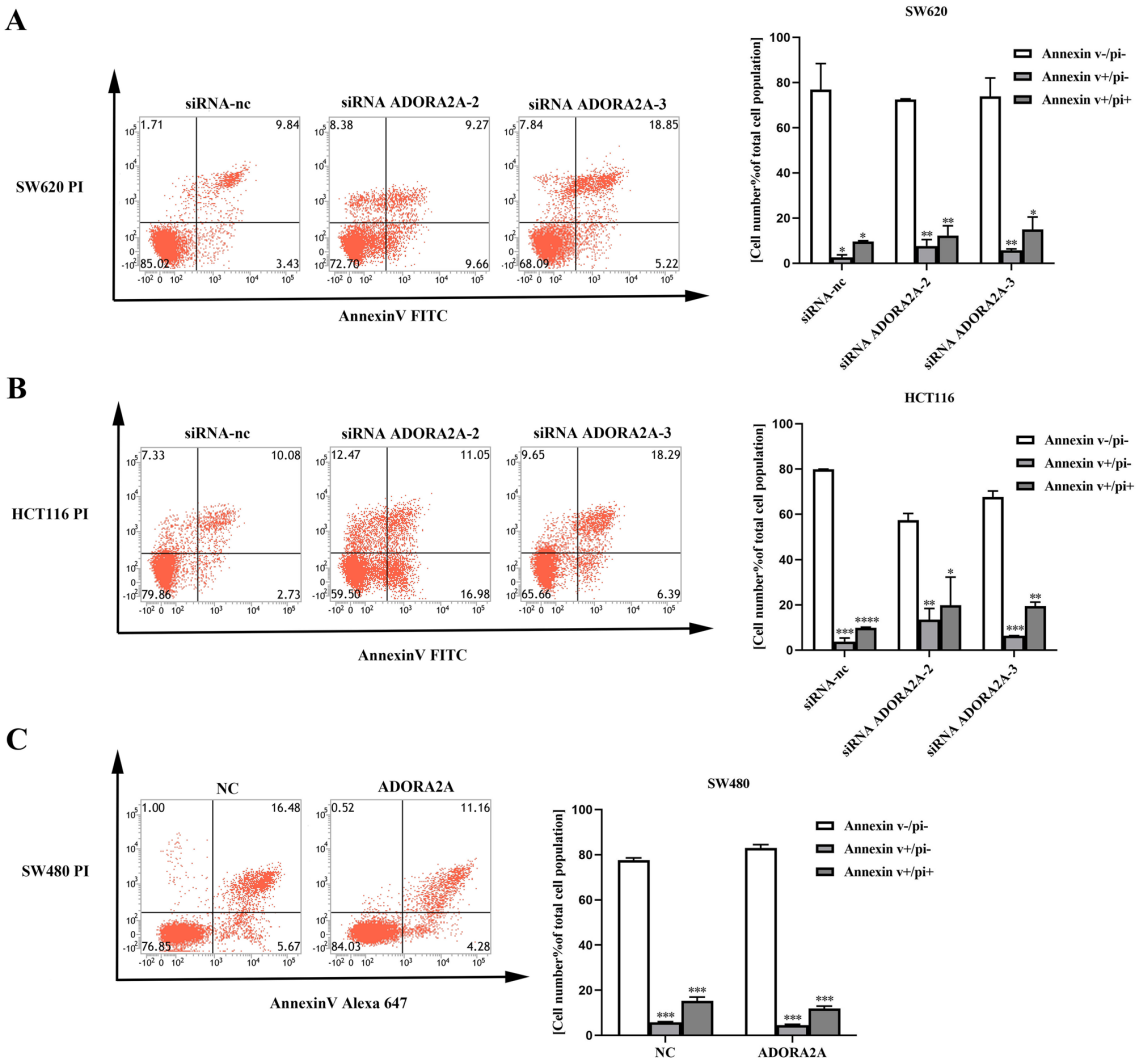
ADORA2A inhibits apoptosis of CRC cells. (**A**–**B**) Cell apoptosis of ADORA2A transfected with siRNA ADORA2A-2 and siRNA ADORA2A-3 in SW620 and HCT116 determined by flow cytometry. Cell apoptosis was calculated as a sum of early and late apoptotic cells. (**C**) Cell apoptosis transfected with ADORA2A overexpression plasmid in SW480 determined by flow cytometry. Cell apoptosis was calculated as a sum of early and late apoptotic cells.

**Figure 6:**
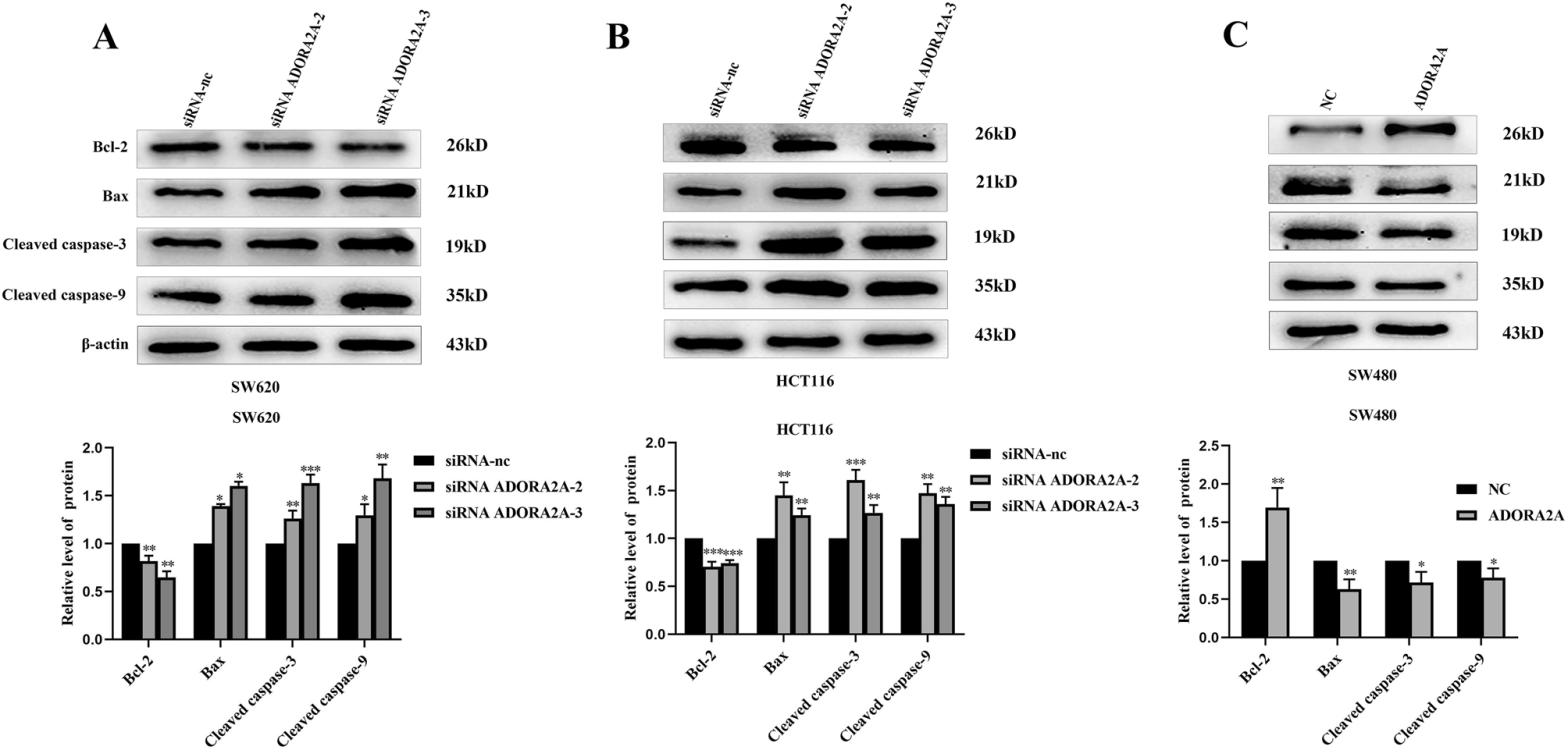
Expression of apoptosis-related proteins after ADORA2A knockdown or over expression. (**A**–**B**) Expression of Bcl-2, Bax, Cleaved caspase-3 and Cleaved caspase-9 in SW620 and HCT116. (**C**) Expression of Bcl-2, Bax, Cleaved caspase-3 and Cleaved caspase-9 proteins in SW480.

We further examined whether ADORA2A contributes to apoptosis by flow cytometry analysis in CRC cells. Compared with NC cells, the apoptosis rate of ADORA2A knockdown cells increased significantly in SW620 and HCT116 (Fig. 5A–B). Meanwhile, the apoptosis rate of overexpression of ADORA2A cells reduced in SW480 (Fig. 5C). In addition, we detected the expression of apoptotic associated proteins. After transfection with siRNA ADORA2A, pro-apoptotic protein expression, primarily Bax, cleaved caspase3 and cleaved caspase9, was up-regulated while anti-apoptotic protein Bcl-2 expression was down-regulated (Fig. 6A–B). In the meantime, overexpression of ADORA2A in SW480 cells showed the opposite results (Fig. 6C). Above all, these results indicated that ADORA2A inhibits apoptosis of CRC cells.

### ADORA2A probably involves in the regulation of PI3K/AKT signaling pathway in CRC

**Figure 7:**
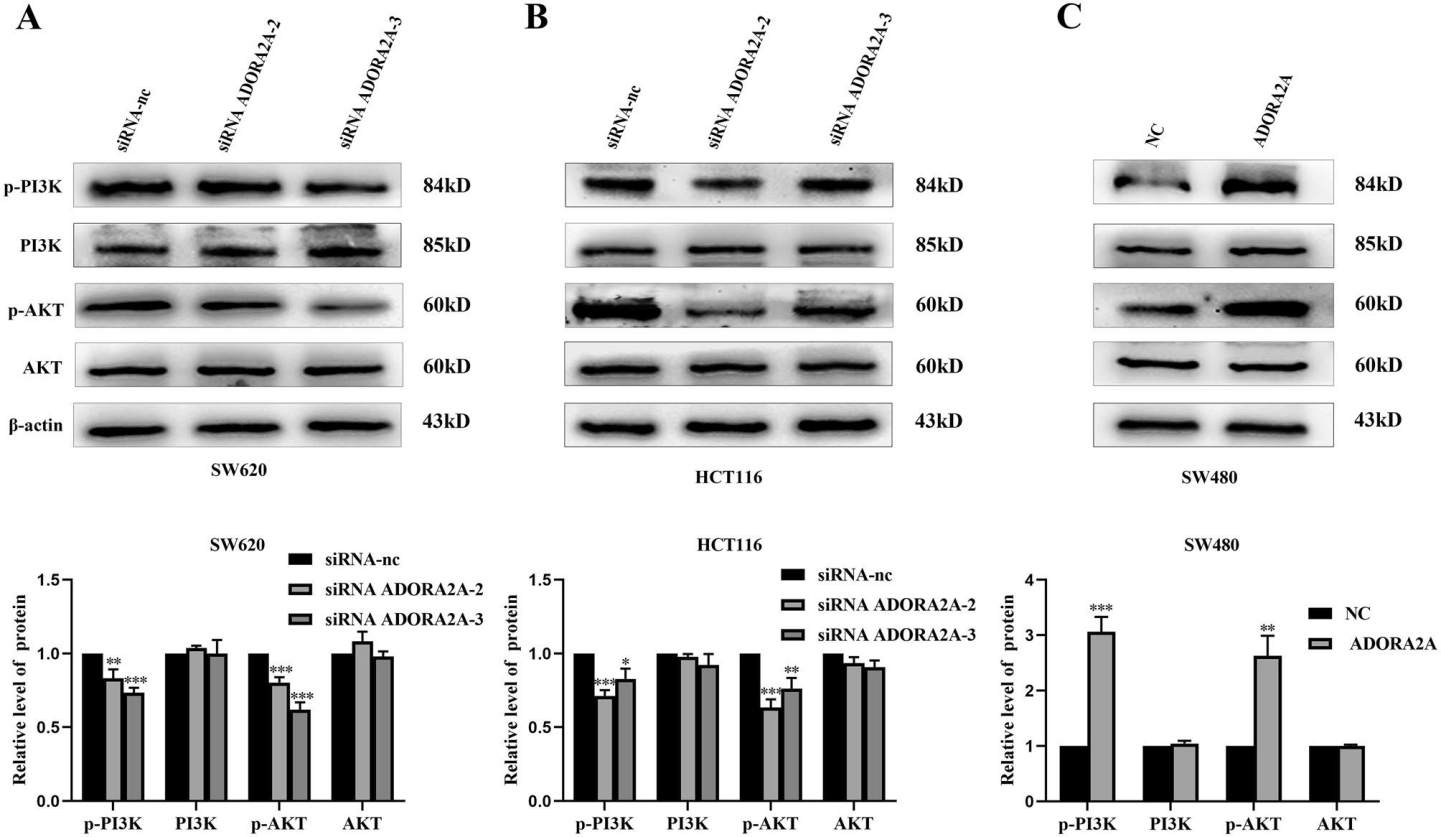
ADORA2A may be involved in the regulation of PI3K/AKT signaling. (**A**–**B**) Proteins expression of p-PI3K, PI3K, p-AKT and AKT in SW620 and HCT116. (**C**) Proteins expression of p-PI3K, PI3K, p-AKT and AKT in SW480.

The PI3K/AKT signaling pathway plays a key regulatory role in cell proliferation and apoptosis^15^. Here, we investigated this ADORA2A-regulated signaling pathway in SW620, HCT116, and SW480 cells. The results showed that p-PI3K and p-AKT levels were decreased in HCT116 and SW620 ADORA2A knockdown cells, while these proteins were increased in SW480 ADORA2A overexpressed cells. However, the expression of PI3K and AKT weren’t changed (Fig. 7A–C). These findings indicated that ADORA2A could regulate the PI3K/AKT signaling pathway in CRC.

## Discussion

ADORA2A, a typical GPCR, participates in many physiopathological processes. Elevated ADORA2A expression was a stand-alone indicator of a good overall survival prognosis in lung cancer patients^16^. ADORA2A /CD73 axis may play a crucial role in the evolution of HNSCC, and ADORA2A hypermethylation was substantially related with poor overall survival^17^. Our results also showed that high ADORA2A expression was associated with poor survival in CRC. Thus, ADORA2A might serve as an indicator of CRC patients.

In addition, as one of the P1 receptors, ADORA2A is involved in the proliferation and invasion of breast cancer^18^. Adenosine Interaction with ADORA2A Promotes Gastric Cancer Metastasis^19^. However, the expression and role of ADORA2A in CRC remains unclear. In this study, ADORA2A was knocked down in SW620 and HCT116 cells, which prevented cell proliferation, migration, and invasion. ADORA2A overexpression encourages the biologically malignant nature of colorectal cancer. These results suggest that colorectal cancer oncogene ADORA2A may affect the prognosis of the disease.

Apoptosis is a crucial step in eliminating the majority of tumor-causing cells for tissue homeostasis^20^. The apoptotic pathways include extrinsic (cytoplasmic) and intrinsic (mitochondrial) pathways^21^. The last pathway to apoptosis involves proteins from the caspase family, which are also necessary to cause apoptosis. Caspase-3 is the main apoptotic protease, and when it is active, downstream apoptosis cannot help but be generated^22^. Caspase-9, as an essential initiator, caspase required for apoptosis signaling, is activated on the apoptosome complex^23^. Numerous studies have demonstrated a strong association between Bcl-2 and Bax levels in CRC. Most Bcl-2 family members have altered expression patterns in CRC tumors, which contributes to disease development and resistance to treatment^24,25^. In this work, we discovered that decreased ADORA2A expression raised the levels of Bax, cleaved caspase-9, and cleaved caspase-3 expression in SW620 and HCT116 cells, but decreased Bcl2 expression when compared to the control group. Additionally, overexpressing ADORA2A in SW480 cells decreased apoptosis. The outcomes showed that ADORA2A can cause apoptosis of CRC cells.

The PI3K/AKT signaling pathway is one of the major signal transduction pathways that promotes cell proliferation and inhibits apoptosis^26^. AKT, sometimes referred to as protein kinase B, is the primary downstream PI3K signaling molecule. AKT that has been activated then controls cell division, differentiation, migration, and glucose metabolism by phosphorylating its downstream target proteins^27,28^. Studies have shown that the PI3K/AKT pathway is crucial for the development of breast, lung, colorectal, and other cancers^29–31^. Previous studies proved that inhibition of the PI3K/AKT pathway is of great significance for cancer treatment^34^. Our research shows that when ADORA2A expression was downregulated in SW620 and HCT116 cells, p-PI3K and p-AKT levels decreased. ADORA2A overexpression in SW480 cells may also promote proliferative, migratory, and invasive traits by upregulating p-PI3K and p-AKT expression, among other malignant features. These findings suggest that ADORA2A may be involved in the control of the PI3K/AKT pathway. However, ADORA2A expression in patients and its relationship to proliferation and apoptosis markers require further verification.

## Conclusion

Overall, it can be said that ADORA2A was very important for the growth, invasion, and apoptosis of CRC cells. Furthermore, the PI3K/AKT signaling pathway could play a role in this process. A promising new target for CRC treatment may be ADORA2A.

## Materials and methods

### RNA-seq

Previous study, we performed RNA-seq with SETDB1 stable knockdown and overexpression cell line HCT116 by Biomarker Technology Corporation (Beijing, China) with log2|fold change|≥ 1.5 and P-value < 0.05^32^. As more DEGs were fund in SETDB1 kncokdown cells. In this study, we focus primarily on RNA-seq data with SETDB1 knockdown.

### Survival analysis

The Human Protein Atlas (HPA, http://www.proteinatlas.org) is a database that maps tissues and organs using information on proteins, transcriptomes, and system biology. HPA pathology database patient records for CRC patients were used for survival analysis.

### Cell culture and transfection

Human CRC cell lines SW620, HCT116, SW480 used in this study were self-stored in the Department of Pathology, Affiliated Hospital of Southwest Medical University. The origin and cultural conditions were described before^32^. Using Lipofectamine 2000, cells were transfected with ADORA2A siRNA or ADORA2A overexpressed plastic after cell density reached around 50%. As a negative control, cells were transfected with siRNA or plastic nonsense (NC). ADORA2A siRNA was designed by Ruibo (Guangzhou, China) and overexpressed plastic was obtained from OBiO Technology (Shanghai, China).

### Real‑time quantitative PCR

Total RNA was extracted from cells after 24 h of transfection using the total RNA simple kit (Tian Gen, China), and then reverse transcribed to cDNA by the Prime Script RT reagent kit with gDNA Eraser (TaKaRa, Japan). TB Green Premix Ex Taq™ (TaKaRa, Japan) was used to express ADORA2A mRNA. After normalization to β-actin, the relative quantification of ADORA2A was calculated using the2^−∆∆Ct^ technique. Primers used were as follows: ADORA2A sense: 5′-TCGCCATTGACCGCTACATTGC-3′, antisense: 5′-AAACGACAGCACCCAGCAGATG-3’, β-actin sense: 5′-CATGTACGTTGCTATCCAGGC-3′, antisense: 5′-CTCCTTAATGTCACGCACGAT-3′.

### Western blot and antibody

For cells that had been transfected for 48 h, ice-cold RIPA buffer (Beyotime, China) containing protease inhibitors and phosphatase inhibitors was used to lyse the cells. (Beyotime, China). The primary antibodies used in this study were as follow: anti-β-actin (1:1000; cat. no. AF0003 Beyotime); anti-ADORA2A (1:2000; cat. no. ab79714 Abcam); anti-Bcl-2 (1:2000; cat. no. 12789-1-AP; Proteintech); anti-Bax (1:1000; cat. no. 50599-2-Ig; Proteintech); anti-caspase3 (1:1000; cat. no. 19677-1-Ap; Proteintech); anti-caspase9 (1:1000; cat. no. 10380-1-Ap Proteintech); anti-p-PI3K (1:1000; cat. no. AF3241; Affinity); anti-PI3K (1:1000; cat. no.AF6241 Affinity); anti-p-AKT (1:2000; cat. no. 4060 T Cell Signaling Tech-nology); anti-AKT (1:1000; cat. no.9272 Cell Signaling Tech-nology). The membrane or the captured image was cropped to remove irrelevant parts and present only the proteins of interest. The cropped image had clear borders or lines to show where the original image was cut. The cropping did not alter or misrepresent the original data, affect the interpretation or quantification of the results, or conceal or manipulate any data. The original, full-length images were kept and documented, and were uploaded in Supplementary Information file. The grayscale of the proteins was measured by the software Image J (NIH Image, Maryland, USA).

### CCK8 assay

CCK8 assay was performed with CCK-8 kit (Beyotime, China). 2000 ~ 2500 cells were transfected for 24 h and placed in a 96-well plate. 100 μl spent medium was replaced with an equivalent volume of new medium containing 10 μl of CCK8 after the cells had been cultivated. The cells were then incubated for 1 h at 37 °C. Using a microplate spectrophotometer, the OD value at 450 nm was measured to calculate the rate of cell growth.

### Colony formation assay

In order to see cell clones, 800 cells were put into a 6 well culture plate and grown for 2 weeks at 37 °C in 5% CO_2_. The clones were stained for 10 min with 1% crystal violet staining solution after being fixed with 4% methanol. An optical microscope was used to assess the number of clones generated.

### Wound healing assay

Approximately 5 × 10^5^ cells were transfected and put onto a 6 well culture plate after 48 h. When the cell density is around 90%, cell scratches are conducted using a 10 μl pipet tip, and the cell debris is entirely removed using RPMI 1640 media. Wells were filled with complete media containing 10% fetal bovine serum and cells were incubated at 37 °C in 5% CO_2_. At 0 h, 24 h, and 48 h, respectively, the microscope camera captured the reduced distance of cell scratches.

### Transwell invasion assay

Cells (1 × 106) that transfected for 24 h, were seeded in the upper chamber of transwell chamber (Corning, USA). 45ul mixed Matrigel (Serum-free medium: Matrigel = 9:1) was added to the transwell chamber (Corning, USA). Transfection after 48 h, the upper chambers of the wells contained 200uL of cell suspension, while the bottom chambers received 600ul of RPMI1640 medium with 30% fetal bovine serum. Before being preserved in methanol for 30 min and staining for 5 min with a 1% crystal violet staining solution, the chambers were cultured for 48 h. Using a microscope camera, cells that migrated to the bottom surface of the membrane were counted and recorded.

### Apoptosis detection

Knockdown cell apoptosis was analyzed using Annexin V-FITC Apoptosis Detection Kit (Dojindo, Japan) and overexpress cell apoptosis was analyzed using Annexin V-Alexa Fluor 647/PI Apoptosis Detection Kit (Yeasen Biotechnology, China). After transfection for 24 h, the cells were harvested and operated according to instructions. Statistical analysis was performed using Flowjo software (Tree Star, Ashland).

### Statistical analysis

Experimental data were analyzed using GraphPad Prism 9.0 software, and statistical analyses were performed using SPSS 26.0. Mean and standard deviation were used to express measured data (SD). The two groups were compared using the Student-Test, and three or more groups were compared using a one-way ANOVA. Statistical significance was determined to be P < 0.05, and the significance shown in *P < 0.05, **P < 0.01, ***P < 0.001 and ****P < 0.0001.

## Supplementary Information

Supplementary Information.

## Data Availability

The dataset generated and analysed in this study may be available from the corresponding author (T.Y.) on reasonable request.

## References

[1] Xi, Y. & Xu, P. Global colorectal cancer burden in 2020 and projections to 2040. *Transl. Oncol.* **14**(10), 101174 (2021). 34243011 10.1016/j.tranon.2021.101174 PMC8273208

[2] Dekker, E. *et al.* Colorectal cancer. *Lancet* **394**(10207), 1467–1480 (2019). 31631858 10.1016/S0140-6736(19)32319-0

[3] Guren, M. G. The global challenge of colorectal cancer. *Lancet Gastroenterol. Hepatol.* **4**(12), 894–895 (2019). 31648973 10.1016/S2468-1253(19)30329-2

[4] Sagaert, X., Vanstapel, A. & Verbeek, S. Tumor heterogeneity in colorectal cancer: What do we know so far?. *Pathobiology* **85**(1–2), 72–84 (2018). 29414818 10.1159/000486721

[5] Sasaki, N. & Clevers, H. Studying cellular heterogeneity and drug sensitivity in colorectal cancer using organoid technology. *Curr. Opin. Genet. Dev.* **52**, 117–122 (2018). 30261425 10.1016/j.gde.2018.09.001

[6] Giuffrida, L. *et al.* CRISPR/Cas9 mediated deletion of the adenosine A2A receptor enhances CAR T cell efficacy. *Nat. Commun.* **12**(1), 3236 (2021). 34050151 10.1038/s41467-021-23331-5 PMC8163771

[7] Dogra, N., Mani, R. J. & Katare, D. P. Protein interaction studies for understanding the tremor pathway in Parkinson’s disease. *CNS Neurol. Disord. Drug Targets* **19**(10), 780–790 (2020). 32888283 10.2174/1871527319666200905115548

[8] Meng, S. X., Wang, B. & Li, W. T. Serum expression of EAAT2 and ADORA2A in patients with different degrees of Alzheimer’s disease. *Eur. Rev. Med. Pharmacol. Sci.* **24**(22), 11783–11792 (2020). 33275249 10.26355/eurrev_202011_23833

[9] Kobold, N. *et al.* ADORA2A polymorphisms influence methotrexate adverse events in rheumatoid arthritis. *Isr. Med. Assoc. J.* **21**(5), 333–338 (2019). 31140226

[10] Huang, J. *et al.* Differential expression of adenosine P1 receptor ADORA1 and ADORA2A associated with glioma development and tumor-associated epilepsy. *Neurochem. Res.* **41**(7), 1774–1783 (2016). 27038930 10.1007/s11064-016-1893-1

[11] Liu, G. *et al.* The adenosine-A2a receptor regulates the radioresistance of gastric cancer via PI3K-AKT-mTOR pathway. *Int. J. Clin. Oncol.* **27**(5), 911–920 (2022). 35122587 10.1007/s10147-022-02123-x

[12] Huang, J., Mao, Q. & Sun, X. Identification of a DNA repair 9-gene signature for the overall survival prediction of pancreatic cancer. *Ann. Diagn. Pathol.* **57**, 151883 (2022). 35123152 10.1016/j.anndiagpath.2021.151883

[13] Fong, L. *et al.* Adenosine 2A receptor blockade as an immunotherapy for treatment-refractory renal cell cancer. *Cancer Discov.* **10**(1), 40–53 (2020). 31732494 10.1158/2159-8290.CD-19-0980 PMC6954326

[14] Li, Z. *et al.* Genomic landscape of microsatellite instability in Chinese tumors: A comparison of Chinese and TCGA cohorts. *Int. J. Cancer* **151**(8), 1382–1393 (2022). 35567574 10.1002/ijc.34119

[15] Wang, X. *et al.* Novel CDKs inhibitors for the treatment of solid tumour by simultaneously regulating the cell cycle and transcription control. *J. Enzyme Inhib. Med. Chem.* **35**(1), 414–423 (2020). 31899991 10.1080/14756366.2019.1705290 PMC6968521

[16] Vogt, T. J. *et al.* Detailed analysis of adenosine A2a receptor (ADORA2A) and CD73 (5’-nucleotidase, ecto, NT5E) methylation and gene expression in head and neck squamous cell carcinoma patients. *Oncoimmunology* **7**(8), e1452579 (2018). 30221045 10.1080/2162402X.2018.1452579 PMC6136855

[17] de Araujo, J. B. *et al.* Targeting the purinergic pathway in breast cancer and its therapeutic applications. *Purinergic Signal* **17**(2), 179–200 (2021). 33576905 10.1007/s11302-020-09760-9 PMC7879595

[18] Shi, L. *et al.* Adenosine interaction with adenosine receptor A2a promotes gastric cancer metastasis by enhancing PI3K-AKT-mTOR signaling. *Mol. Biol. Cell* **30**(19), 2527–2534 (2019). 31339445 10.1091/mbc.E19-03-0136 PMC6743355

[19] Ashkenazi, A. Directing cancer cells to self-destruct with pro-apoptotic receptor agonists. *Nat. Rev. Drug Discov.* **7**(12), 1001–1012 (2008). 18989337 10.1038/nrd2637

[20] Ghobrial, I. M., Witzig, T. E. & Adjei, A. A. Targeting apoptosis pathways in cancer therapy. *CA Cancer J. Clin.* **55**(3), 178–194 (2005). 15890640 10.3322/canjclin.55.3.178

[21] Ko, H. M. *et al.* Liver-wrapping, nitric oxide-releasing nanofiber downregulates cleaved caspase-3 and bax expression on rat hepatic ischemia-reperfusion injury. *Transplant. Proc.* **49**(5), 1170–1174 (2017). 28583550 10.1016/j.transproceed.2017.03.054

[22] Wurstle, M. L., Laussmann, M. A. & Rehm, M. The central role of initiator caspase-9 in apoptosis signal transduction and the regulation of its activation and activity on the apoptosome. *Exp. Cell Res.* **318**(11), 1213–1220 (2012). 22406265 10.1016/j.yexcr.2012.02.013

[23] Ramesh, P. & Medema, J. P. BCL-2 family deregulation in colorectal cancer: potential for BH3 mimetics in therapy. *Apoptosis* **25**(5–6), 305–320 (2020). 32335811 10.1007/s10495-020-01601-9 PMC7244464

[24] van der Heijden, M. *et al.* Bcl-2 is a critical mediator of intestinal transformation. *Nat. Commun.* **7**, 10916 (2016). 26956214 10.1038/ncomms10916 PMC4786877

[25] Wang, M., Zhang, J. & Gong, N. Role of the PI3K/Akt signaling pathway in liver ischemia reperfusion injury: A narrative review. *Ann. Palliat. Med.* **11**(2), 806–817 (2022). 35016518 10.21037/apm-21-3286

[26] Dornan, G. L. *et al.* Defining how oncogenic and developmental mutations of PIK3R1 alter the regulation of class IA phosphoinositide 3-kinases. *Structure* **28**(2), 145–156 (2020). 31831213 10.1016/j.str.2019.11.013

[27] Uko, N. E. *et al.* Akt Pathway inhibitors. *Curr. Top. Med. Chem.* **20**(10), 883–900 (2020). 32091335 10.2174/1568026620666200224101808

[28] Wang, F. *et al.* Syringin exerts anti-breast cancer effects through PI3K-AKT and EGFR-RAS-RAF pathways. *J. Transl. Med.* **20**(1), 310 (2022). 35794555 10.1186/s12967-022-03504-6 PMC9258109

[29] Jin, Y. *et al.* Activation of PI3K/AKT pathway is a potential mechanism of treatment resistance in small cell lung cancer. *Clin. Cancer Res.* **28**(3), 526–539 (2022). 34921019 10.1158/1078-0432.CCR-21-1943

[30] Yin, F., Huang, X. & Xuan, Y. Pyrroline-5-carboxylate reductase-2 promotes colorectal cancer progression via activating PI3K/AKT/mTOR pathway. *Dis. Mark.* **2021**, 9950663 (2021). 10.1155/2021/9950663 PMC8429024 34512817

[31] He, Y. *et al.* Targeting PI3K/Akt signal transduction for cancer therapy. *Signal Transduct. Target Ther.* **6**(1), 425 (2021). 34916492 10.1038/s41392-021-00828-5 PMC8677728

[32] Xu, D. *et al.* C5aR1 promotes the progression of colorectal cancer by EMT and activating Wnt/β-catenin pathway. *Clin. Transl. Oncol.* **225**, 440–446 (2022). 10.1007/s12094-022-02956-y PMC9873742 36192575

