# Correction: ADORA2A promotes proliferation and inhibits apoptosis through PI3K/AKT pathway activation in colorectal carcinoma

**DOI:** 10.1038/s41598-025-30221-z

**Published:** 2025-12-09

**Authors:** Longyan Ran, Xiao Mou, Zhenglin Peng, Xiaochen Li, Meirong Li, Duo Xu, Zixi Yang, Xingwang Sun, Tao Yin

**Affiliations:** 1 College of Basic Medicine, Southwest Medical University, Luzhou, Sichuan China; 2 College of Clinical Medicine, Southwest Medical University, No.25 Taiping Street, Jiangyang District, Luzhou City, Sichuan China; 3 Department of Pathology, Affiliated Hospital of Southwest Medical University, Luzhou, Sichuan China; 4 Present Address: Luzhou Key Laboratory of Precision Pathology Diagnosis for Serious Diseases, Luzhou, Sichuan China

Correction to: *Scientific Reports* 10.1038/s41598-023-46521-1, published online 09 November 2023

The original version of this Article contained errors in the Figures.

In Figure 3E, as a result of a file labelling error, inappropriate images were selected for the 0h and 24h conditions. In Figure 3G, inappropriate image selection resulted in a partial overlap in the siRNA ADORA2A-2 and siRNA ADORA2A-3 conditions in SW620 cells. In Figure 4D, the NC condition mistakenly showed partial overlap with the ADORA2A condition. In Figure 5, the graphs contained an error in the calculation of cell apoptosis.

The original Figures 3, 4 and 5 and accompanying legends appear below.

**Fig. 3:**
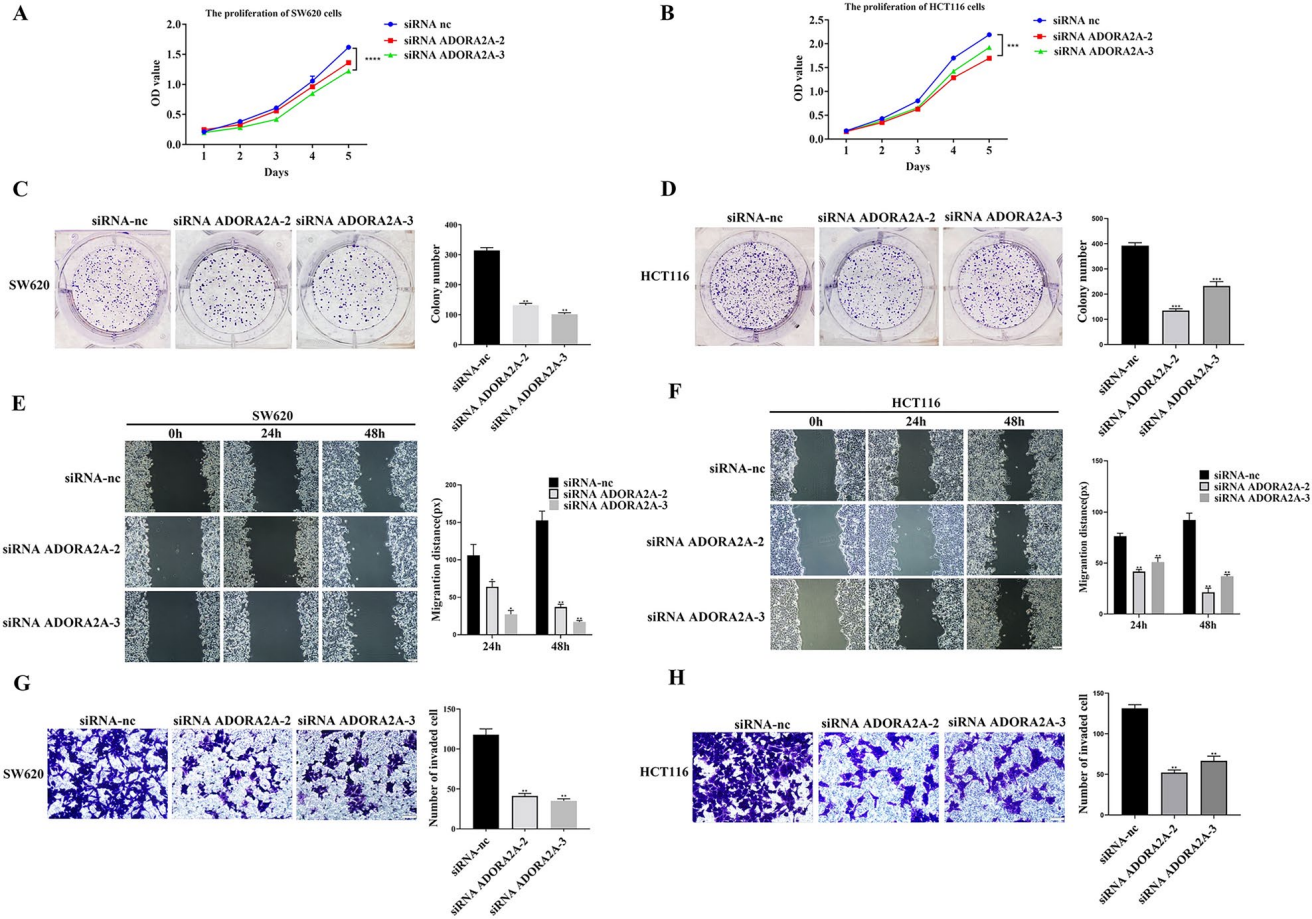
Knockdown of ADORA2A inhibits proliferation, migration, and invasion of CRC cells. (**A**–**B**) CCK8 assay of ADORA2A transfected with siRNA ADORA2A-2 and siRNA ADORA2A-3 in SW620 and HCT116. (**C**–**D**) Colony formation assay of ADORA2A transfected with siRNA ADORA2A-2 and siRNA ADORA2A-3 in SW620 and HCT116. (**E**–**F**) ADORA2A wound healing assay transfected with siRNA ADORA2A-2 and siRNA ADORA2A-3 in SW620 and HCT116. (**G**-**H**) Transwell assay of ADORA2A transfected with siRNA ADORA2A-2 and siRNA ADORA2A-3 in SW620 and HCT116. Bars:200 μm (**E**–**F**); 50 μm (**G**–**H**).

**Fig. 4:**
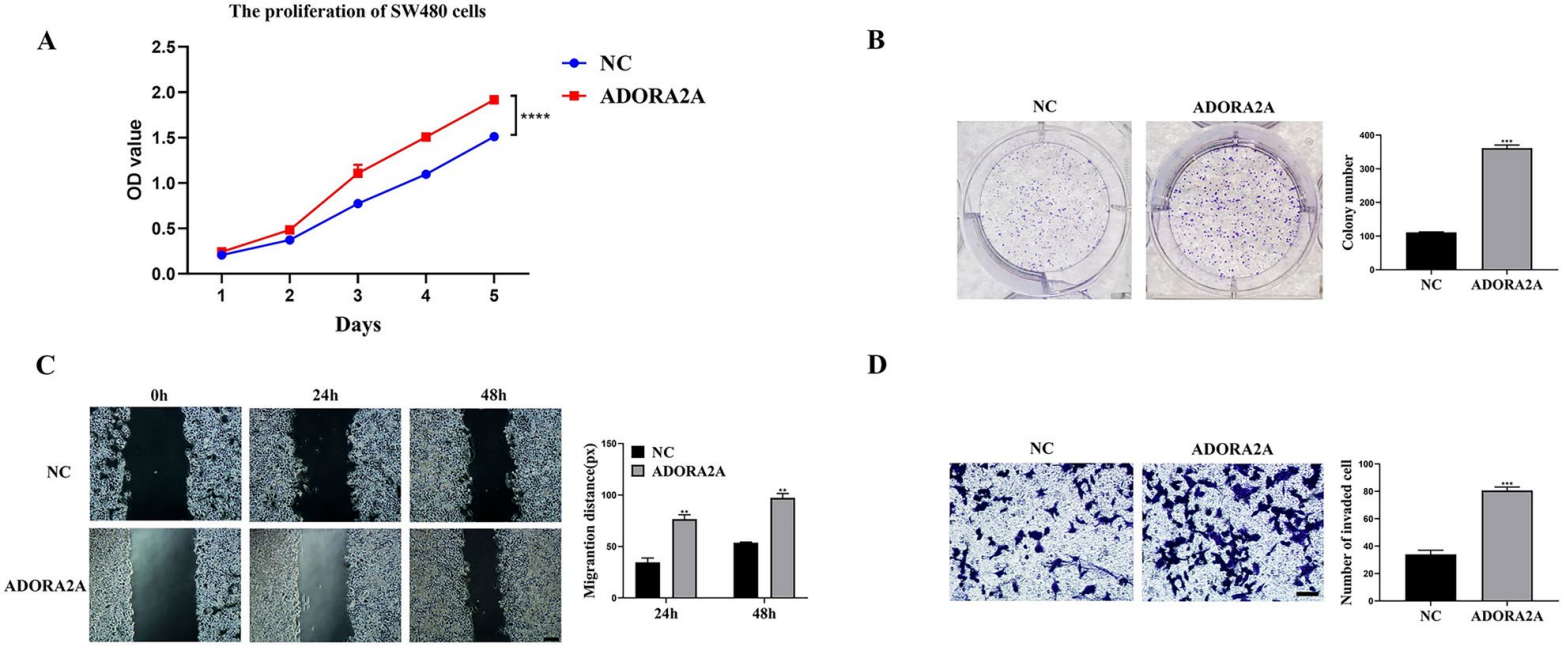
Overexpression of ADORA2A promotes malignant phenotypes in SW480 cells. (**A**–**B**) CCK8 assay and colony formation assay of SW480 cells transfected with ADORA2A overexpression plasmid. (**C**) Wound healing assay of SW480 cells transfected with ADORA2A overexpression plasmid. (**D**) Transwell assay of SW480 cells transfected with ADORA2A overexpression plasmid. Bars:200 μm (**C**); 50 μm (**D**).

**Fig. 5:**
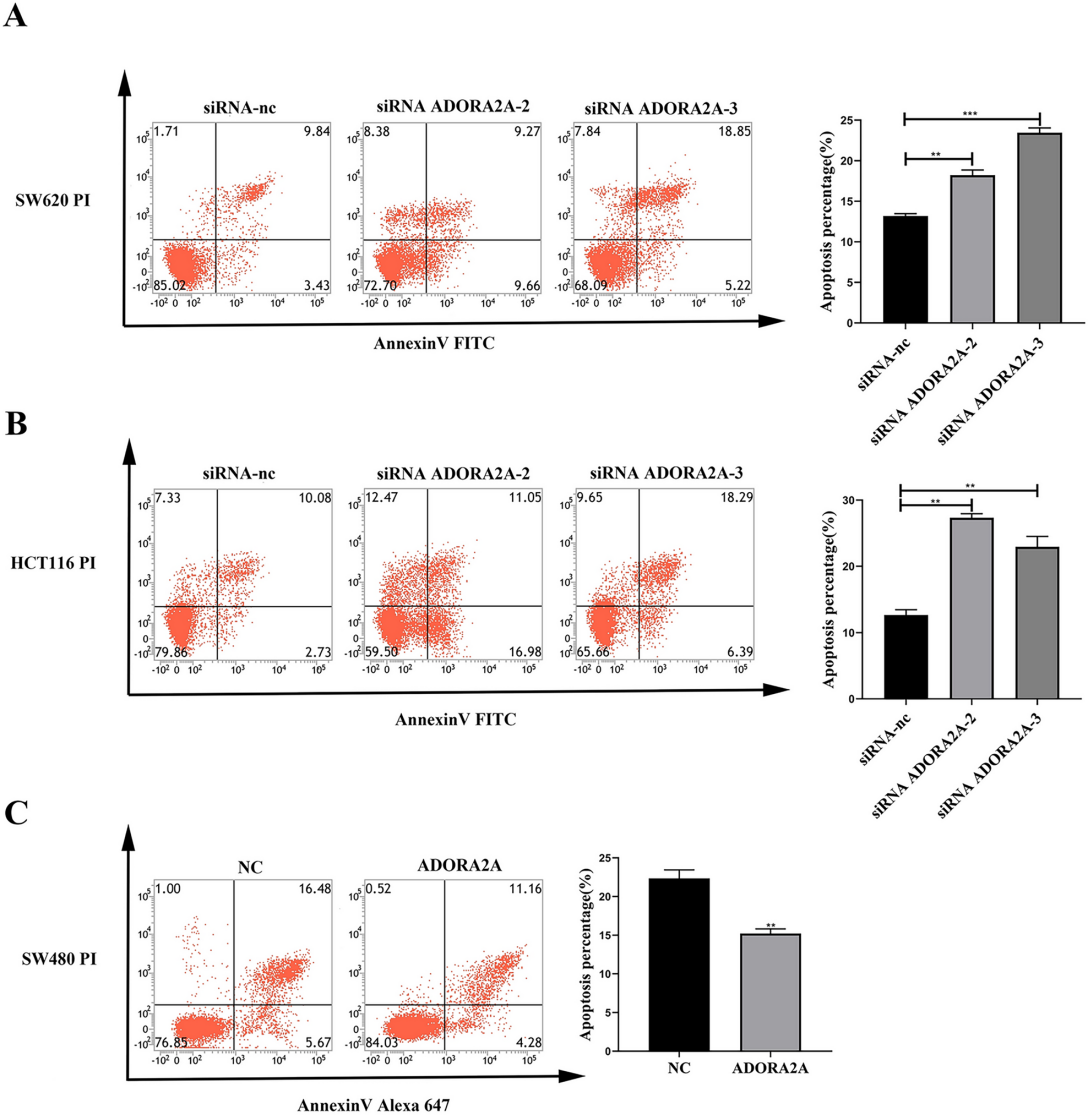
ADORA2A inhibits apoptosis of CRC cells. (**A**–**B**) Cell apoptosis of ADORA2A transfected with siRNA ADORA2A-2 and siRNA ADORA2A-3 in SW620 and HCT116 determined by flow cytometry. Cell apoptosis was calculated as a sum of early and late apoptotic cells. (**C**) Cell apoptosis transfected with ADORA2A overexpression plasmid in SW480 determined by flow cytometry. Cell apoptosis was calculated as a sum of early and late apoptotic cells.

The original Article has been corrected.

