# Supplementary material for: ADORA2A promotes proliferation and inhibits apoptosis through PI3K/AKT pathway activation in colorectal carcinoma
Source: Sci Rep. 2023 Nov 9;13:19477. doi: 10.1038/s41598-023-46521-1 (PMC10636200; doi:10.1038/s41598-023-46521-1)

For Figure 1C

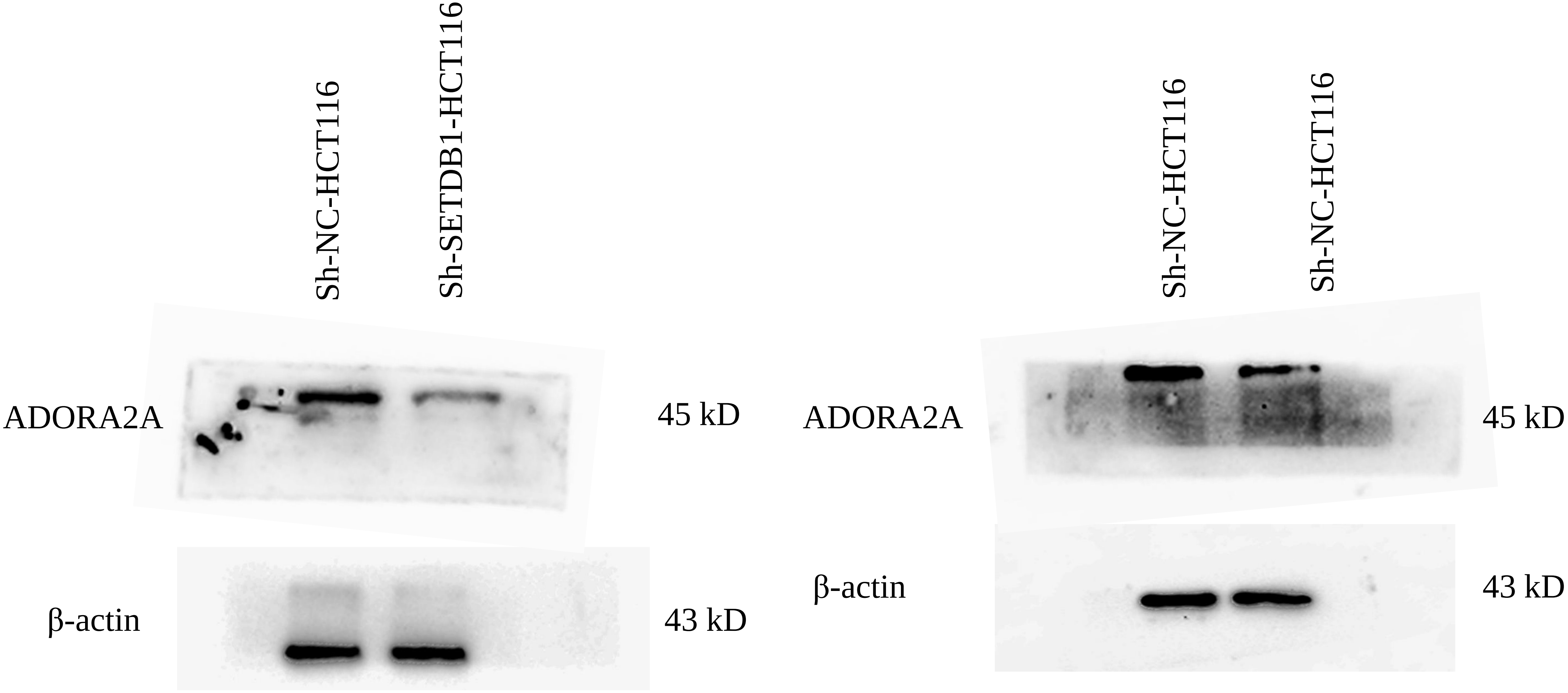

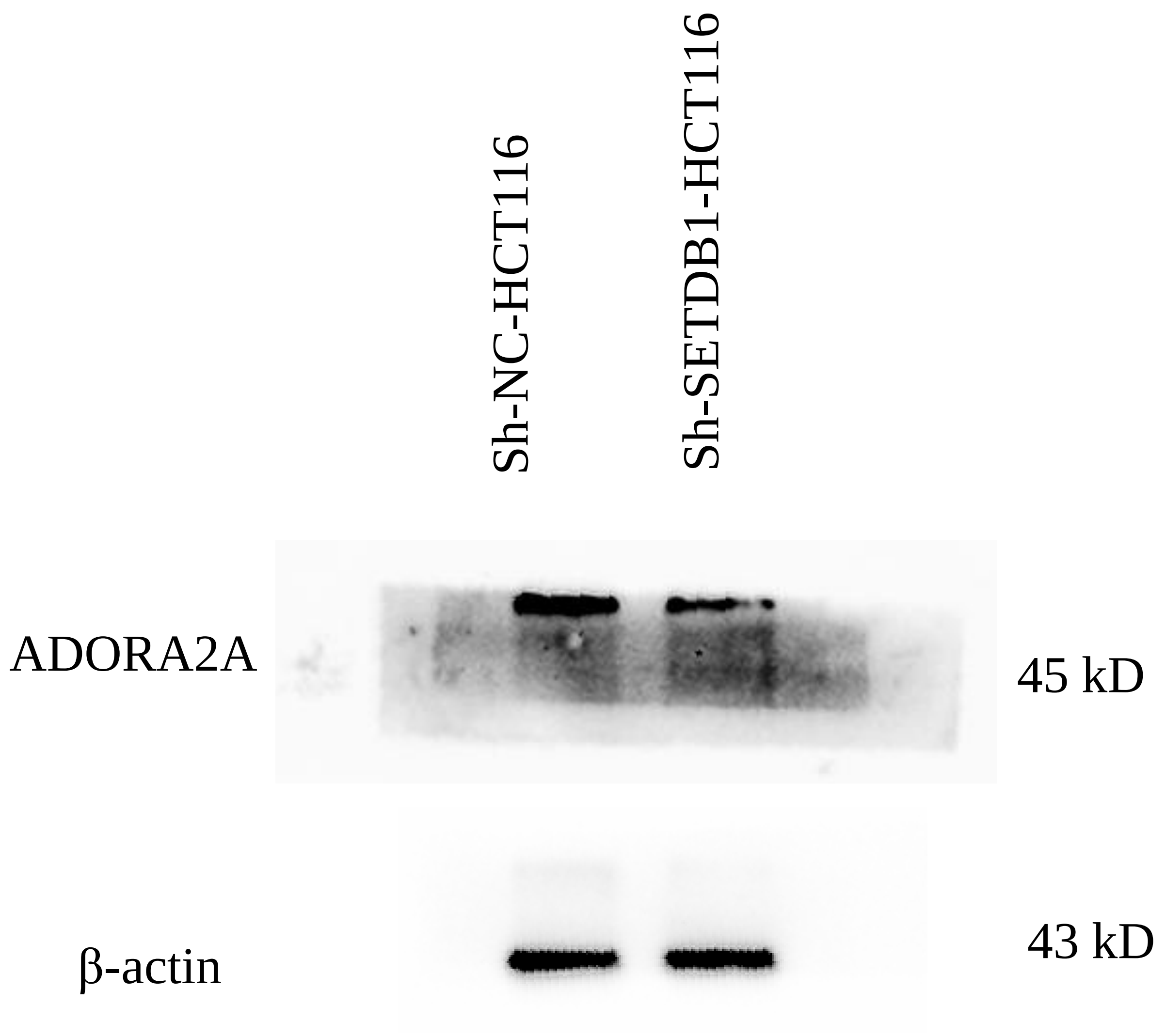
Sh-NC-HCT116
Sh-SETDB1-HCT116
ADORA2A
45 kD
β-actin
43 kD

For Figure 2A

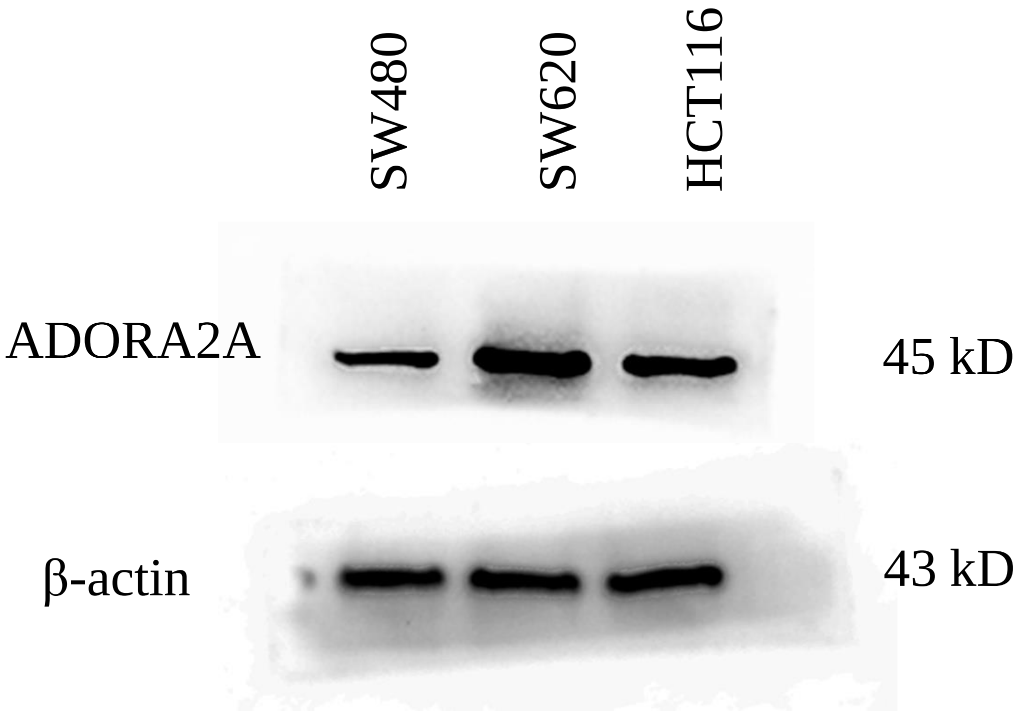


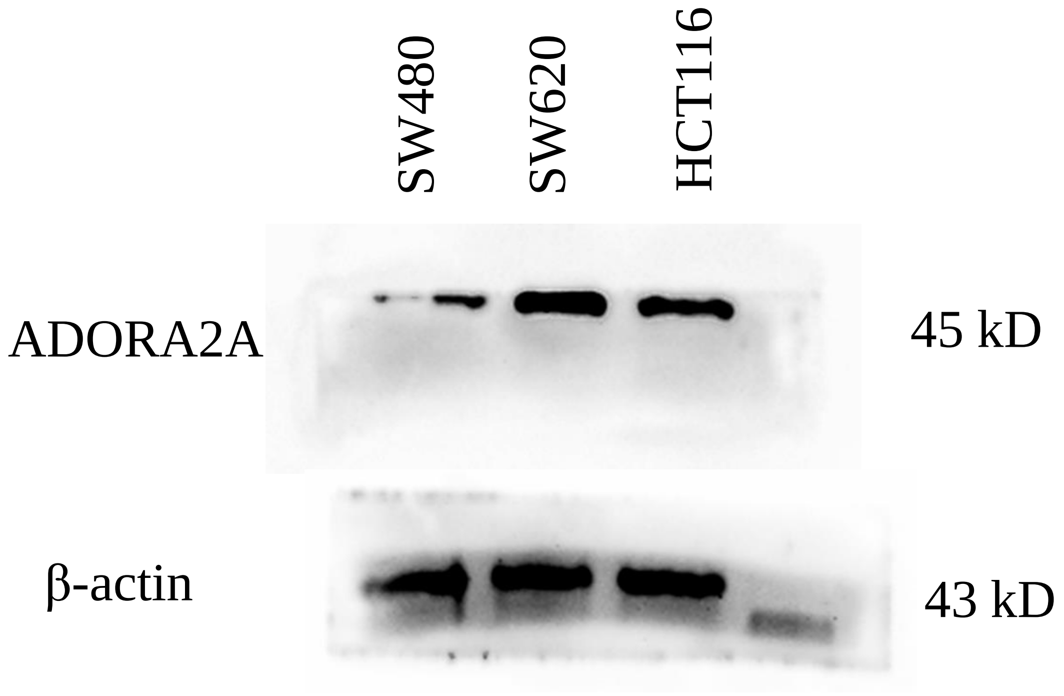

For Figure 2B

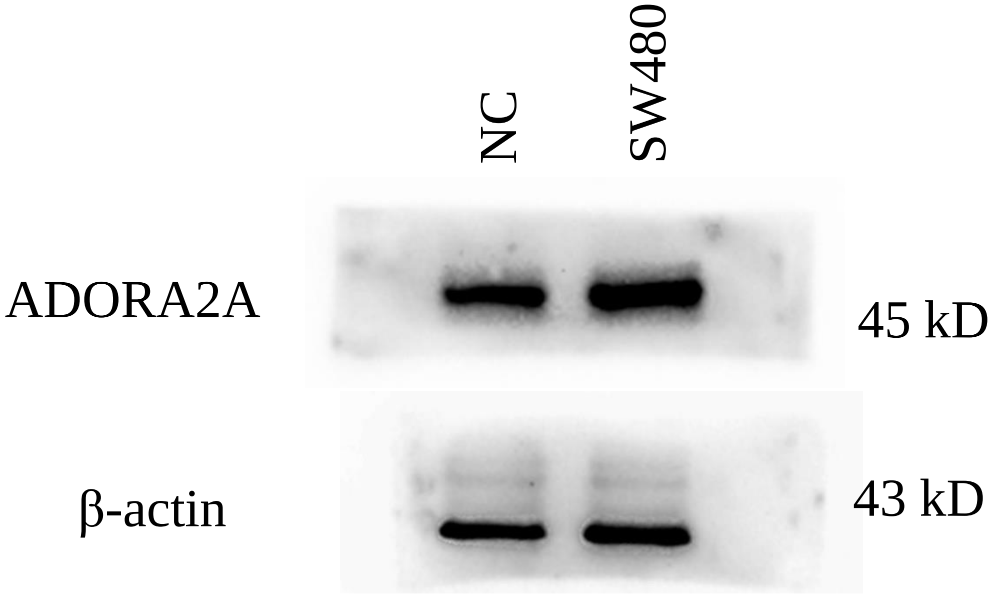


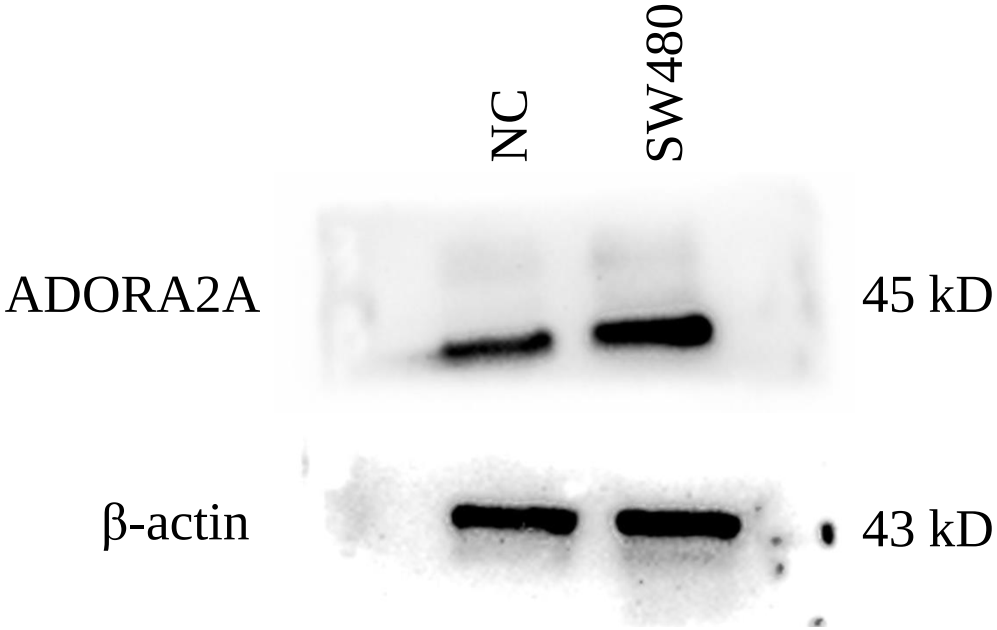

For Figure 2C

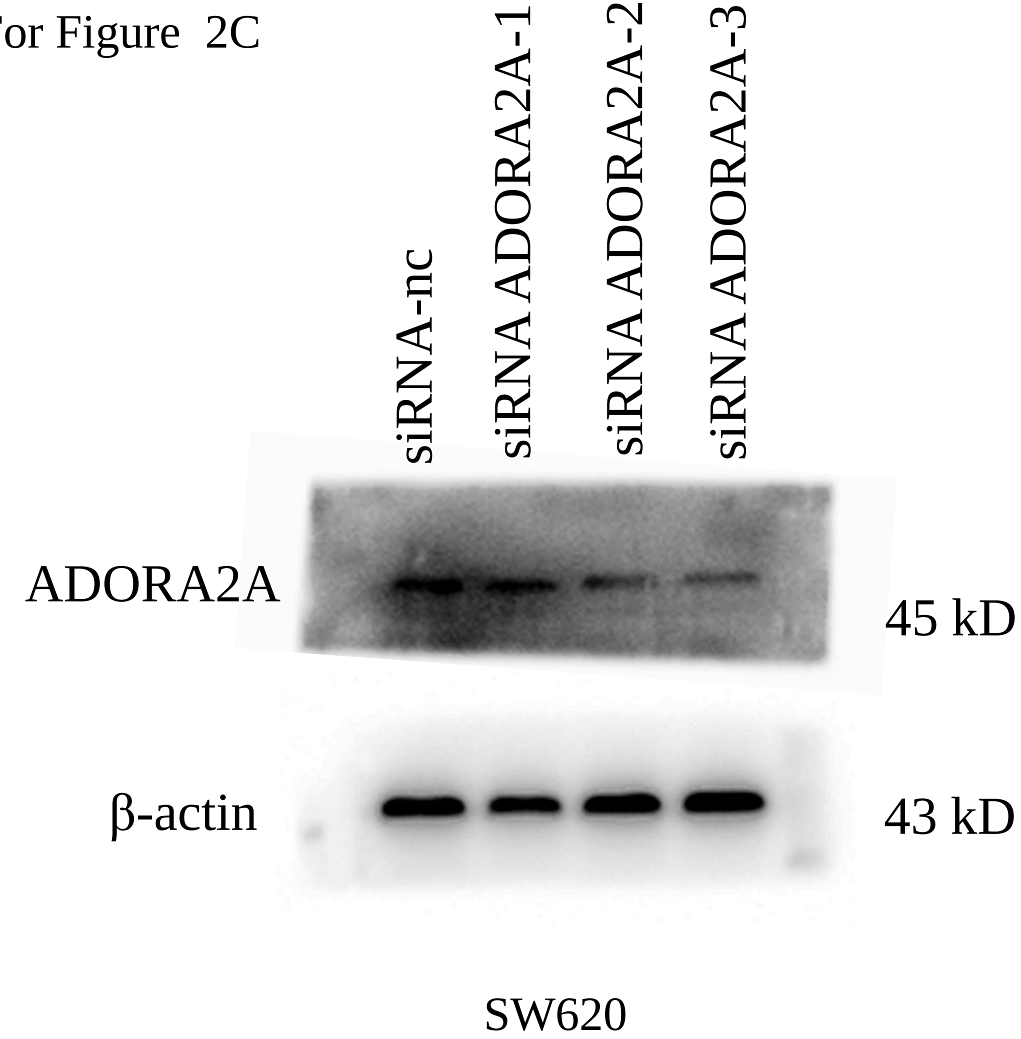


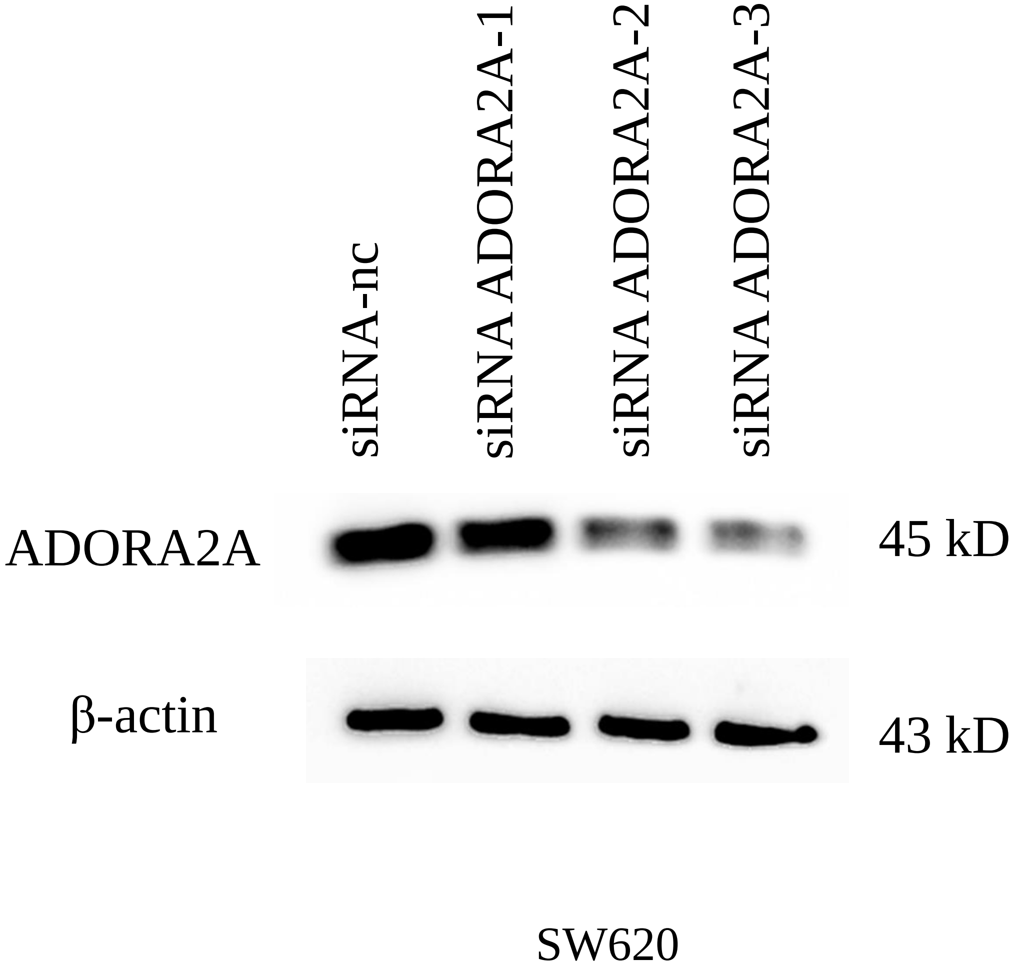

For Figure 2D

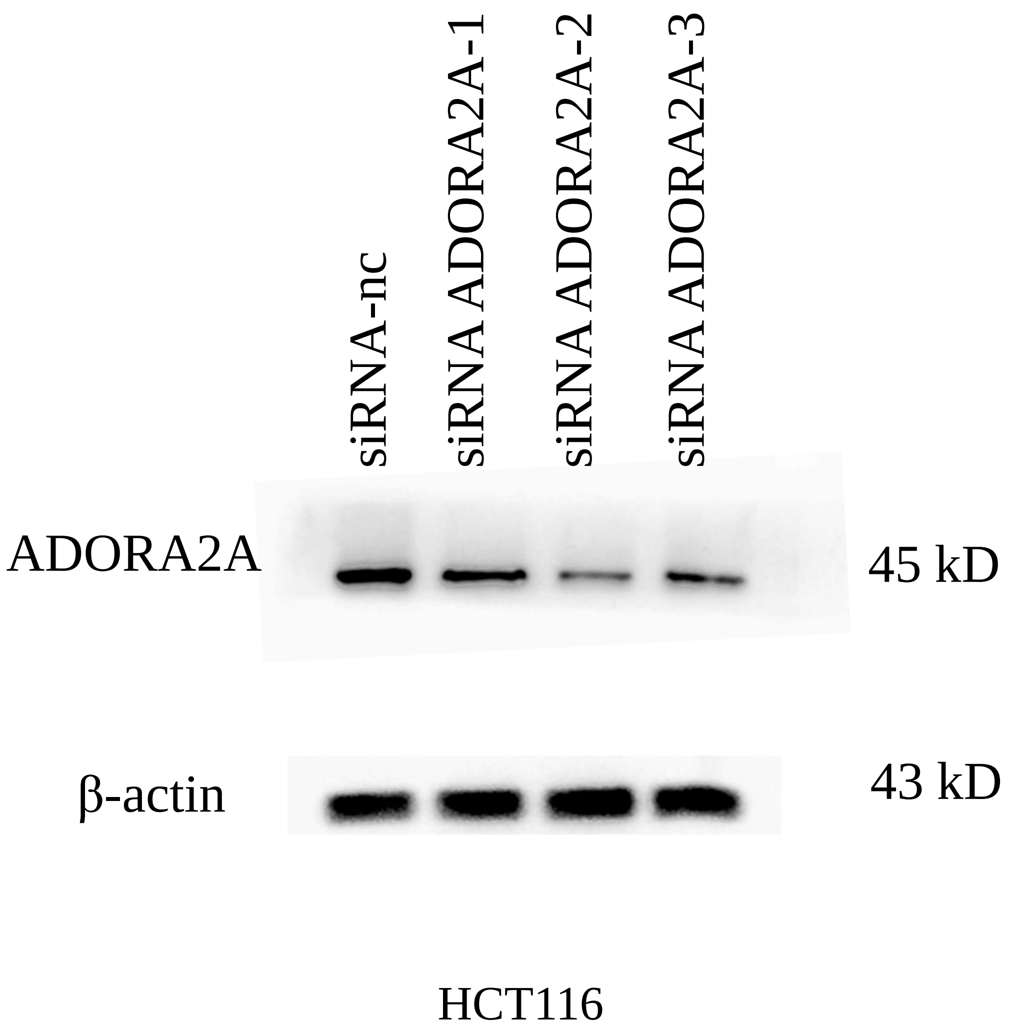


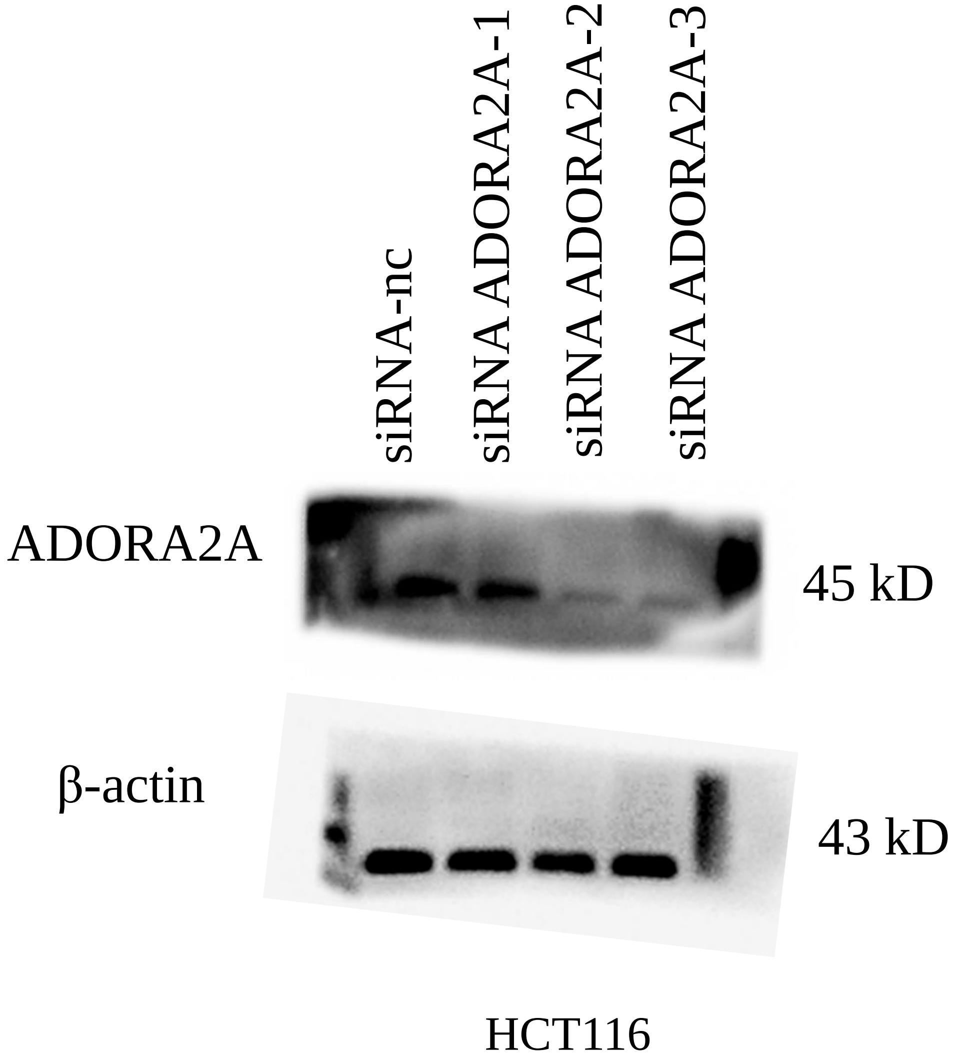

For Figure 6A

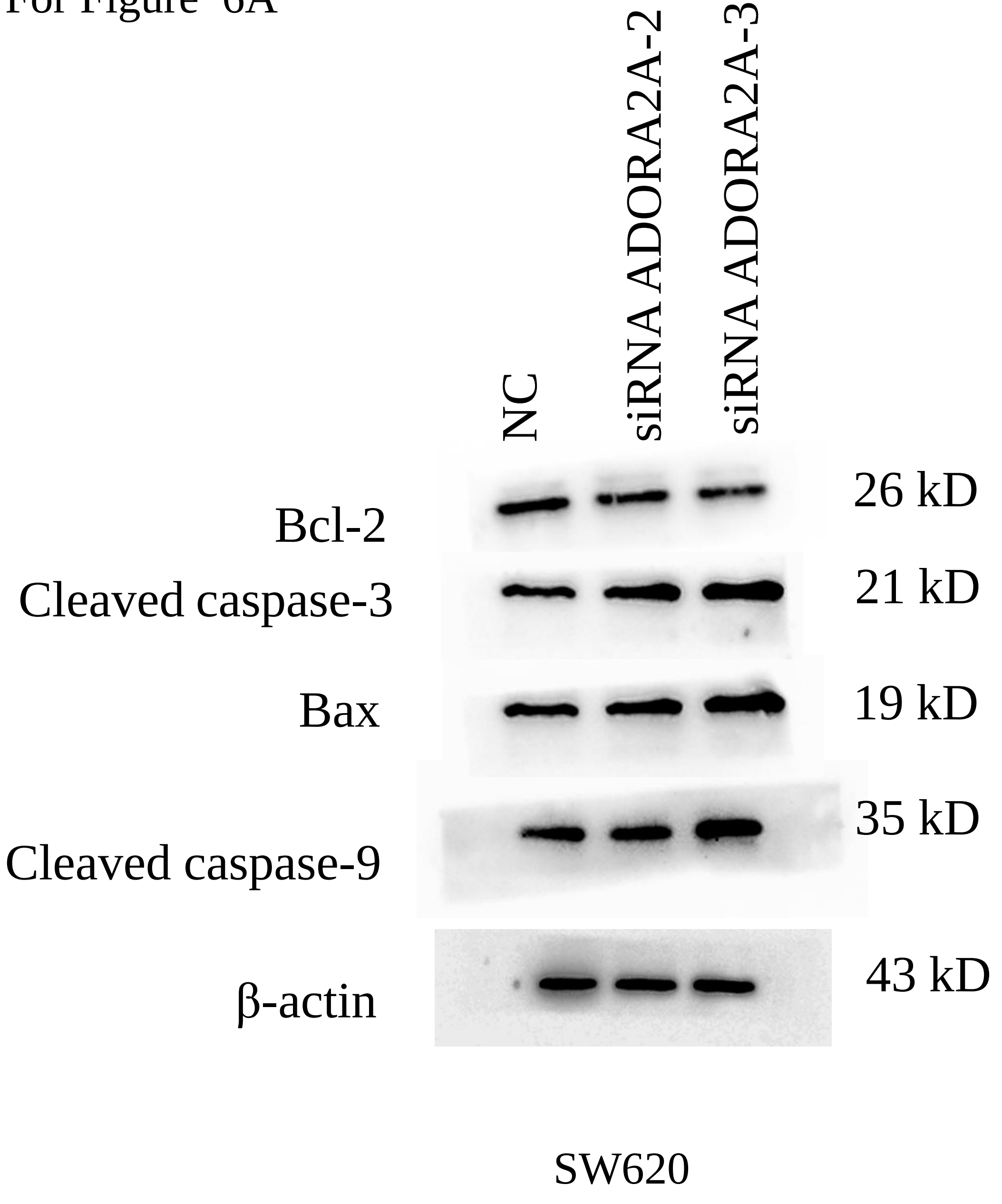


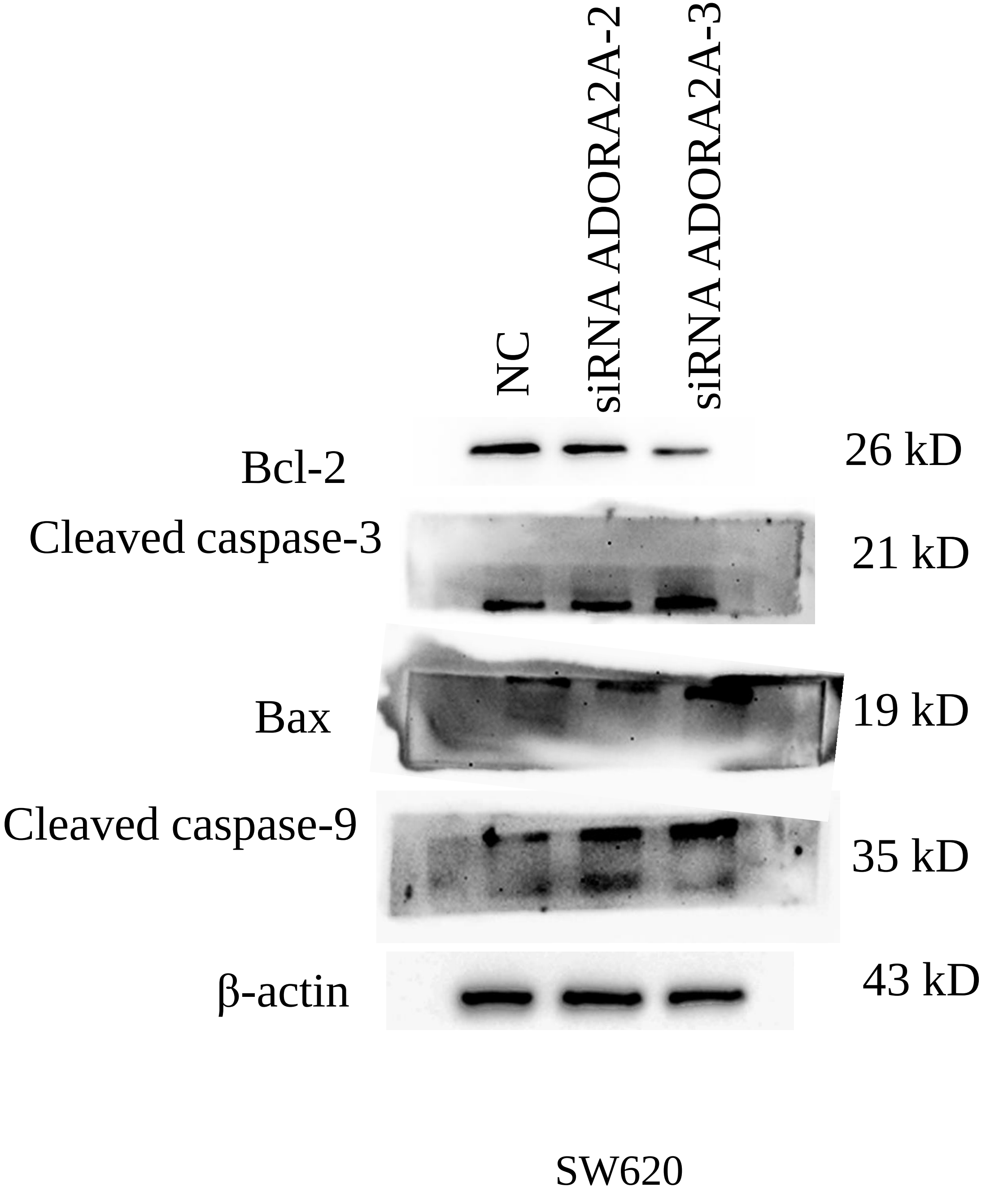

For Figure 6B

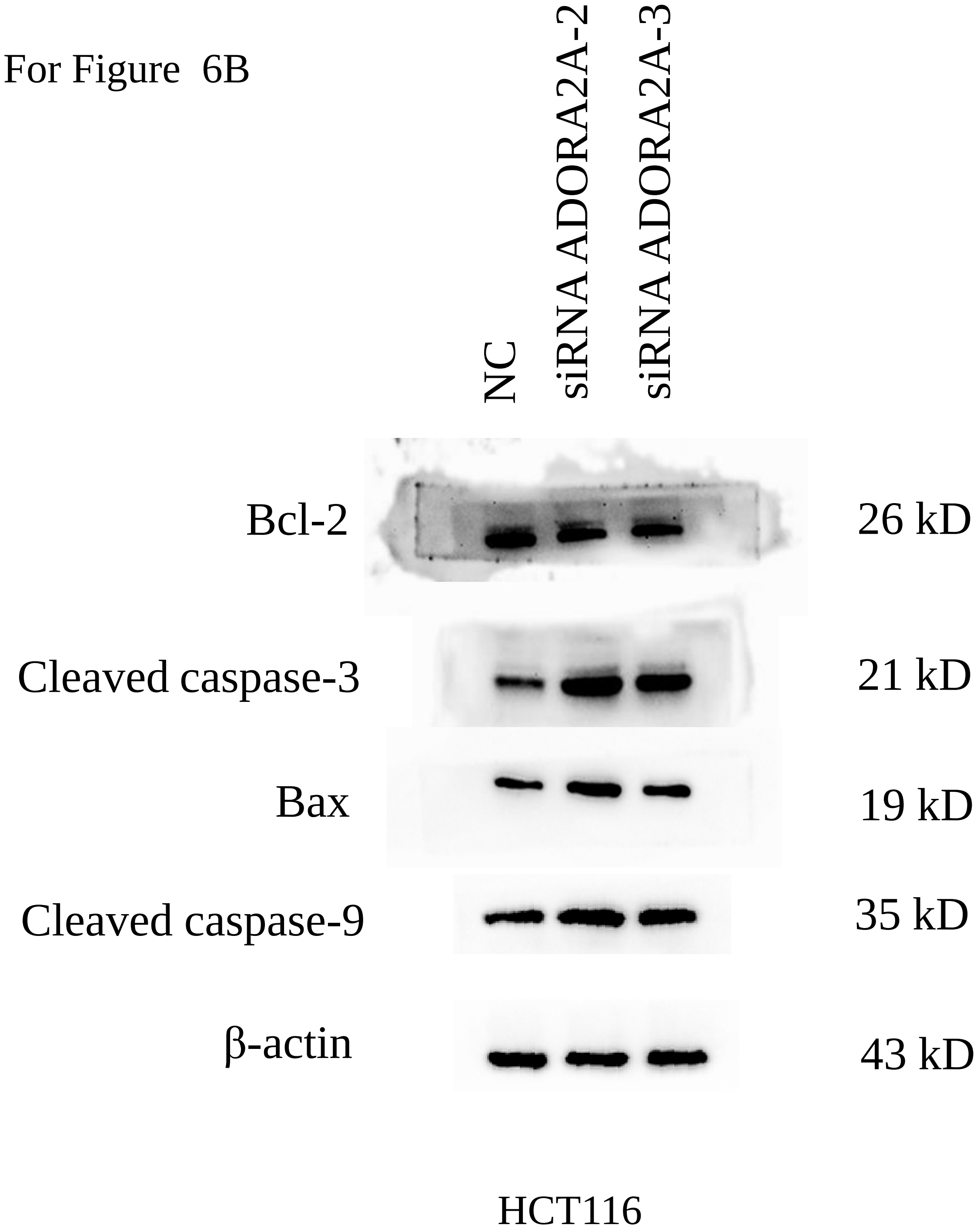


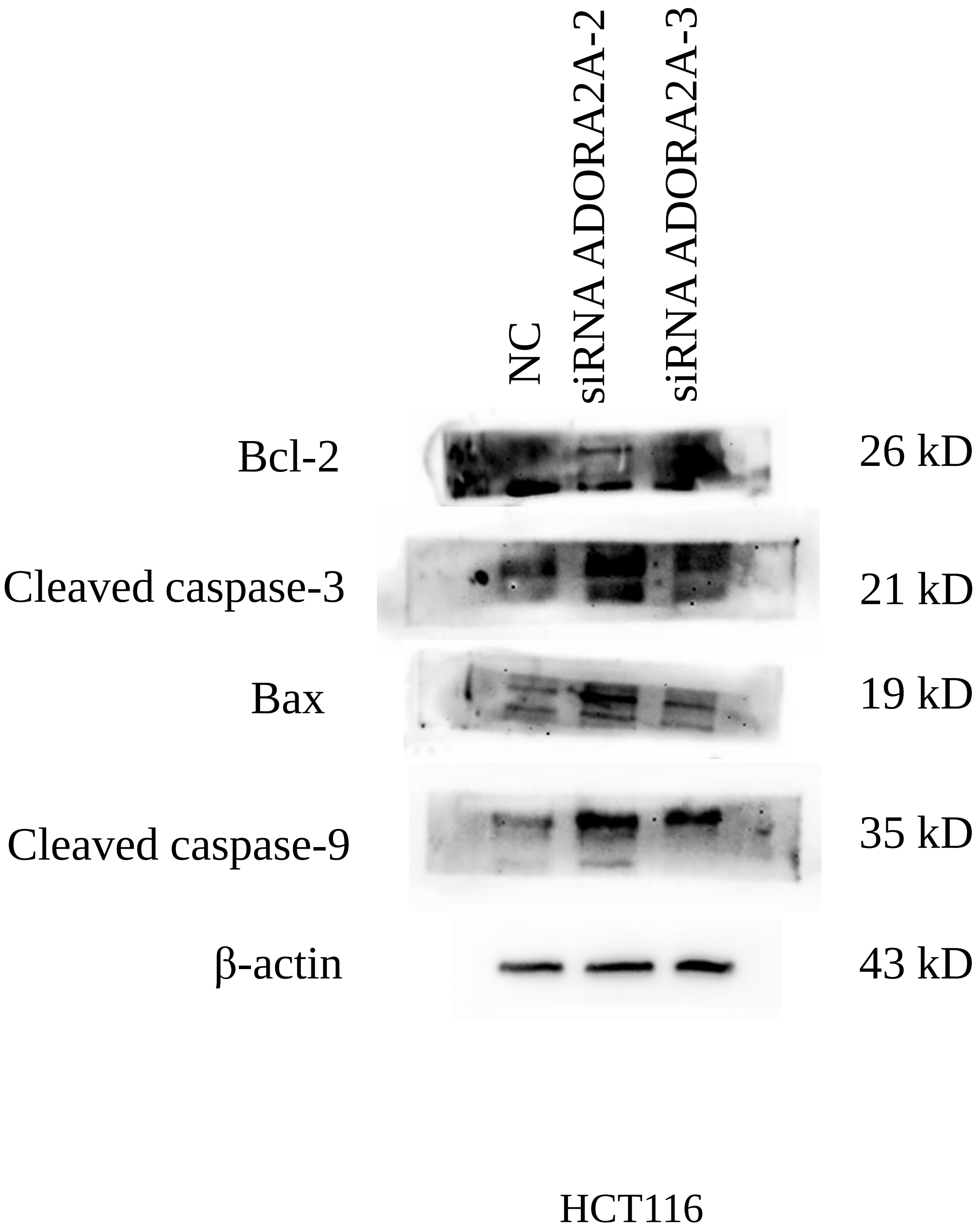

For Figure 6C

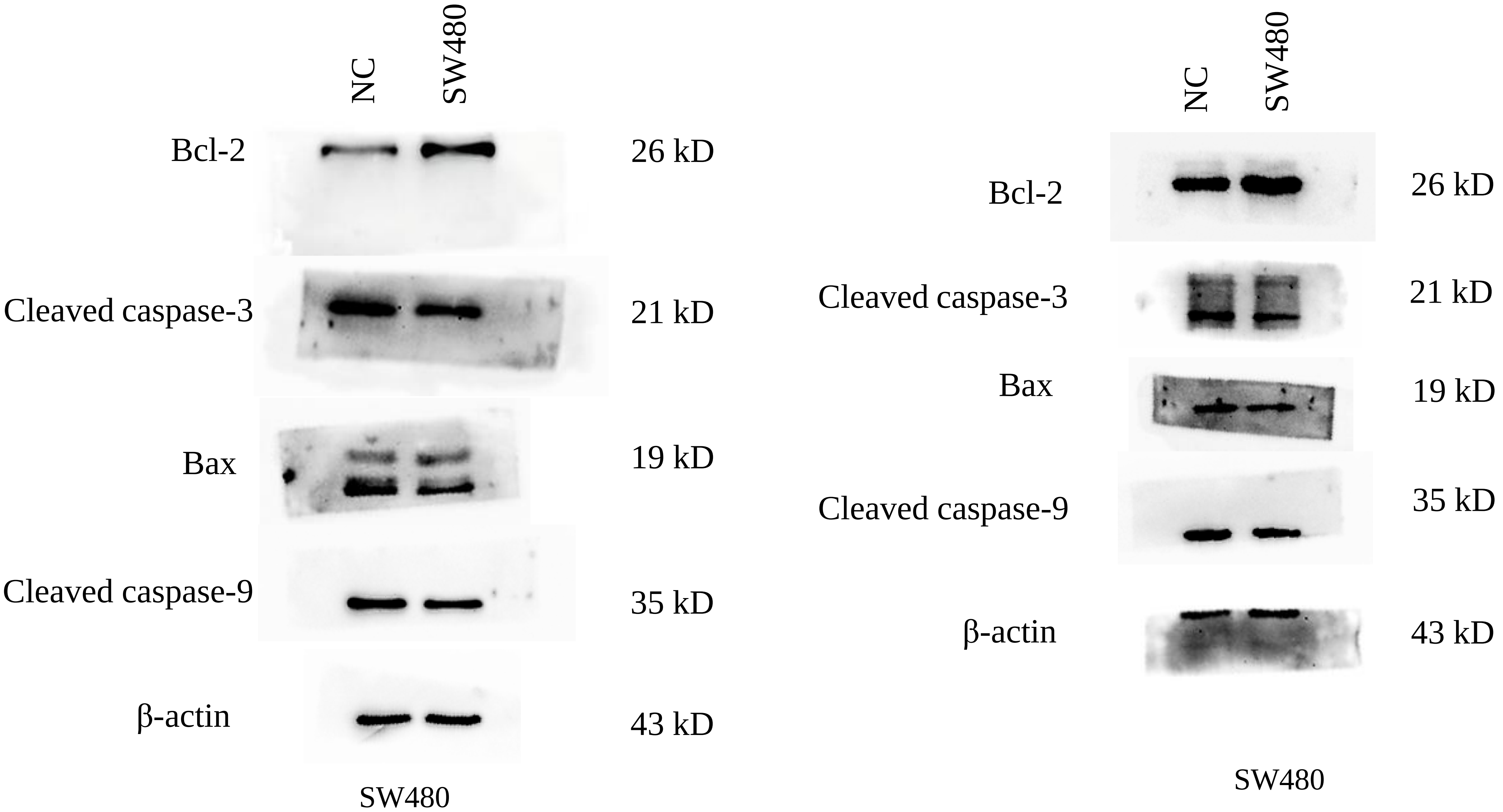

For Figure 7A

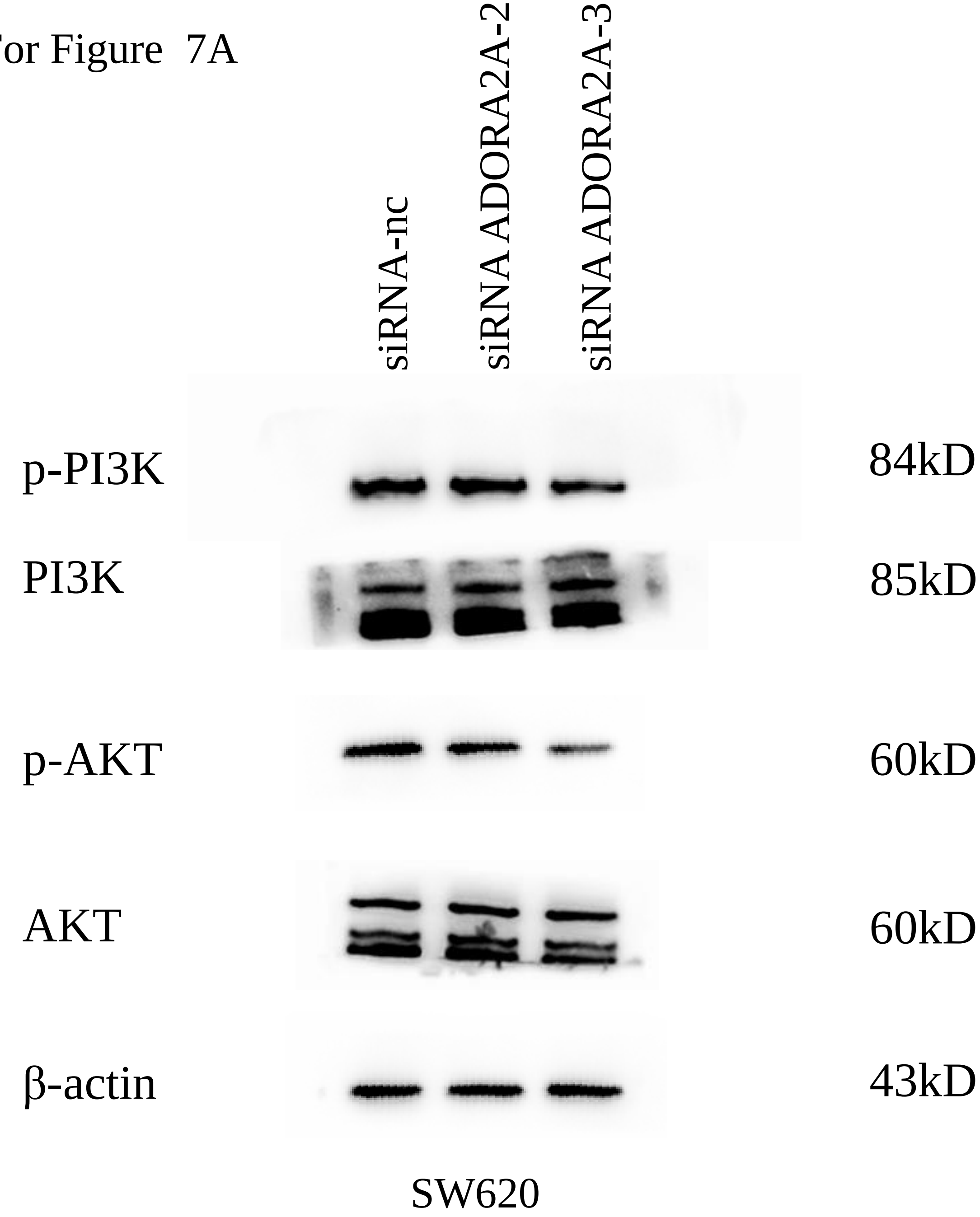


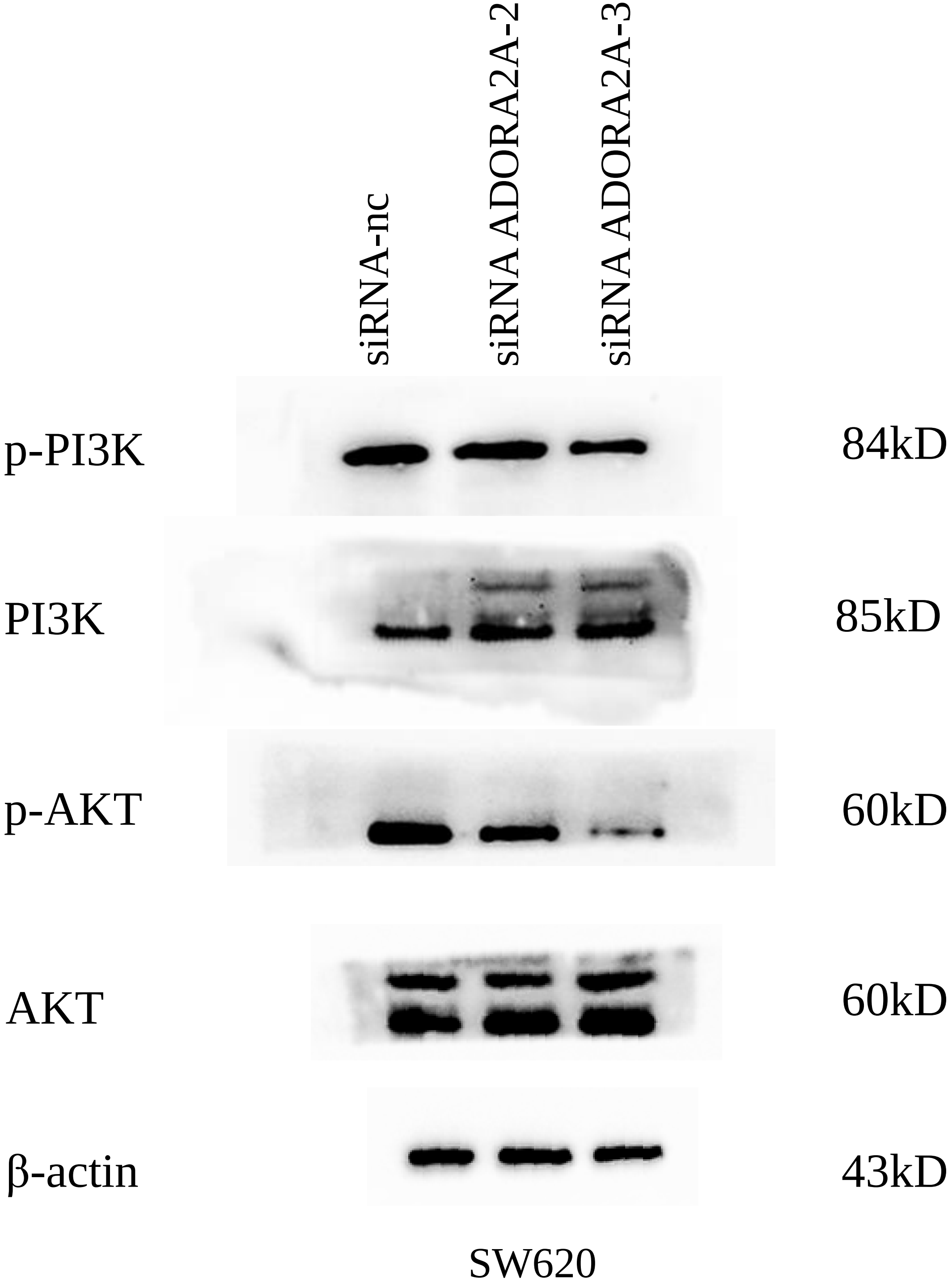

For Figure 7B

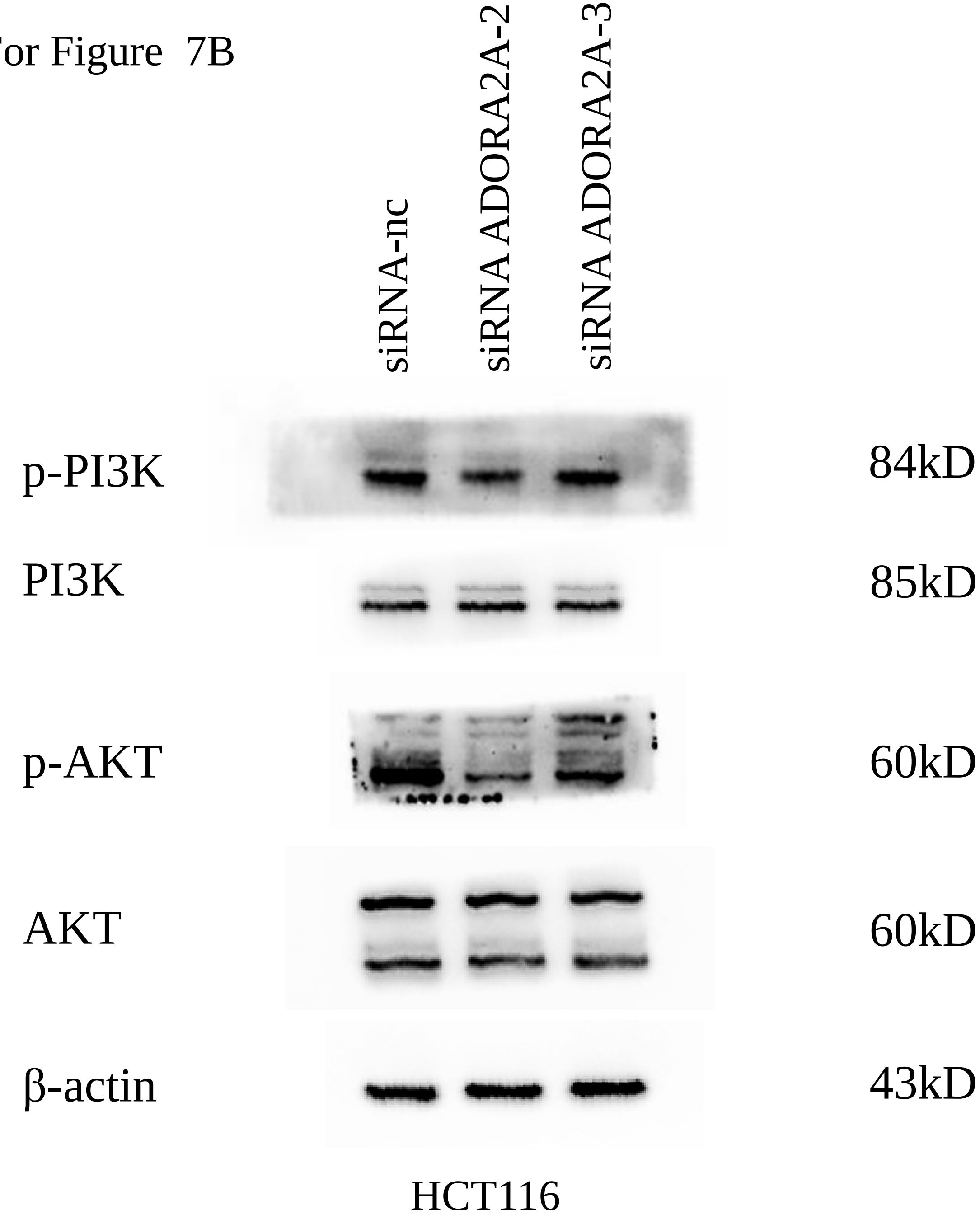


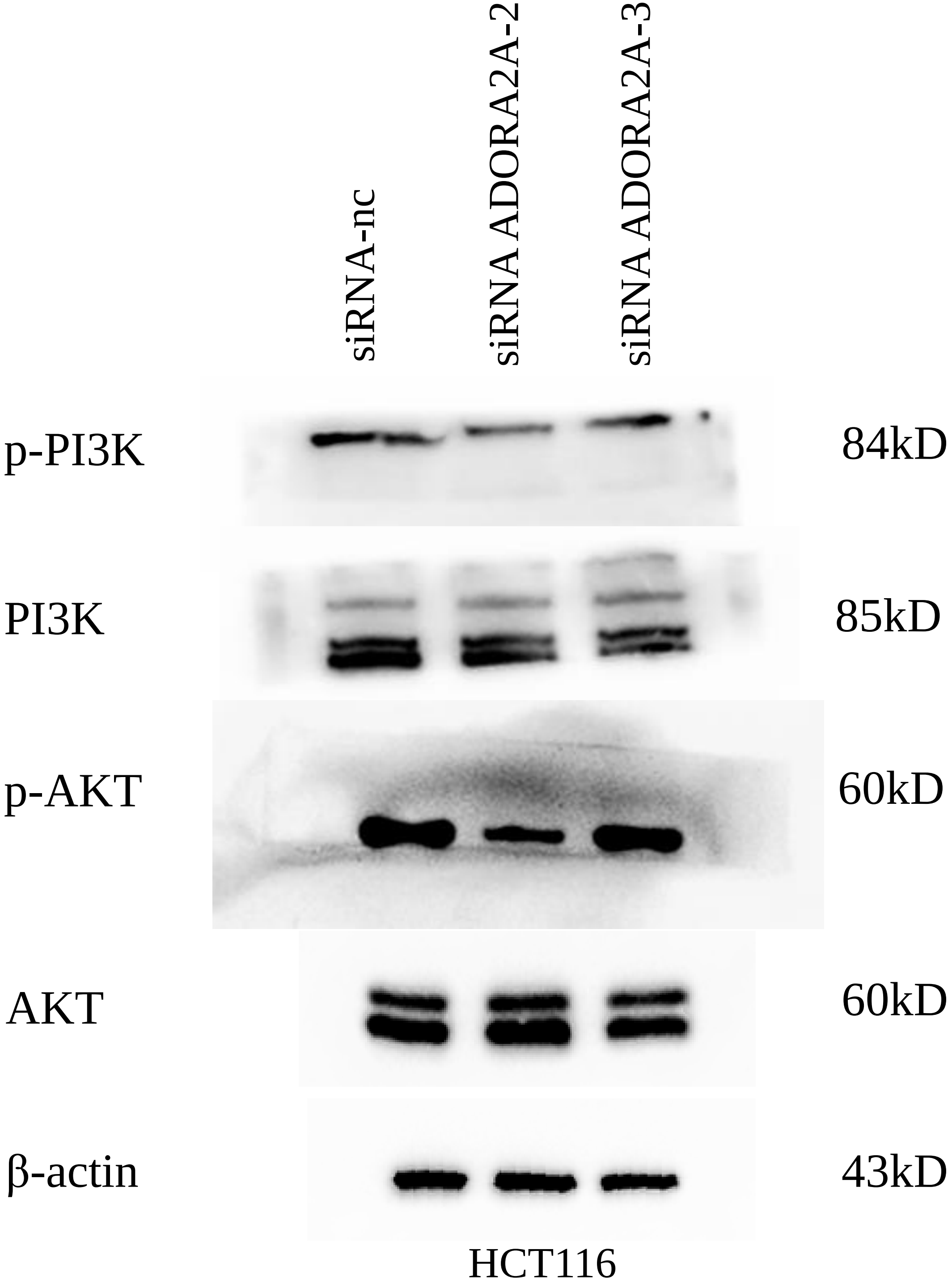

For Figure 7C

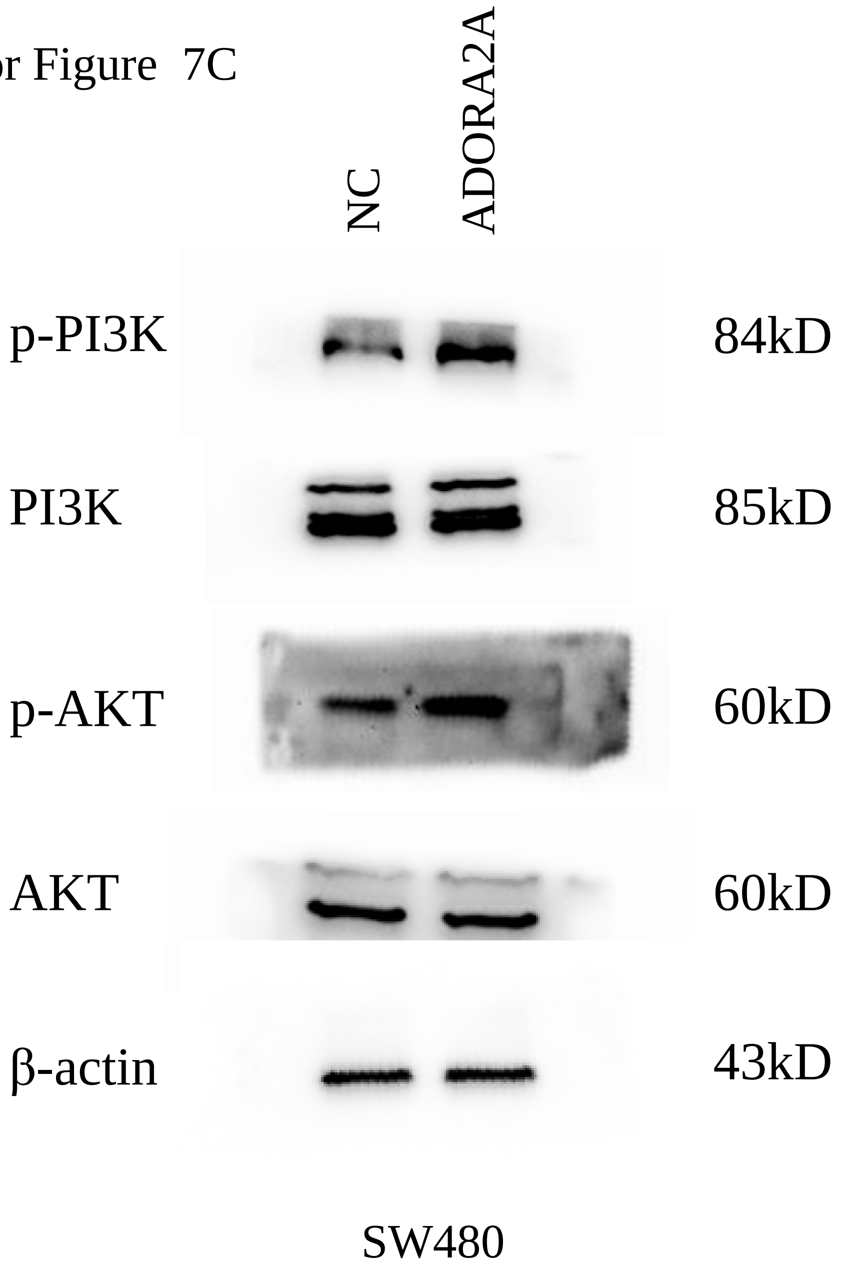


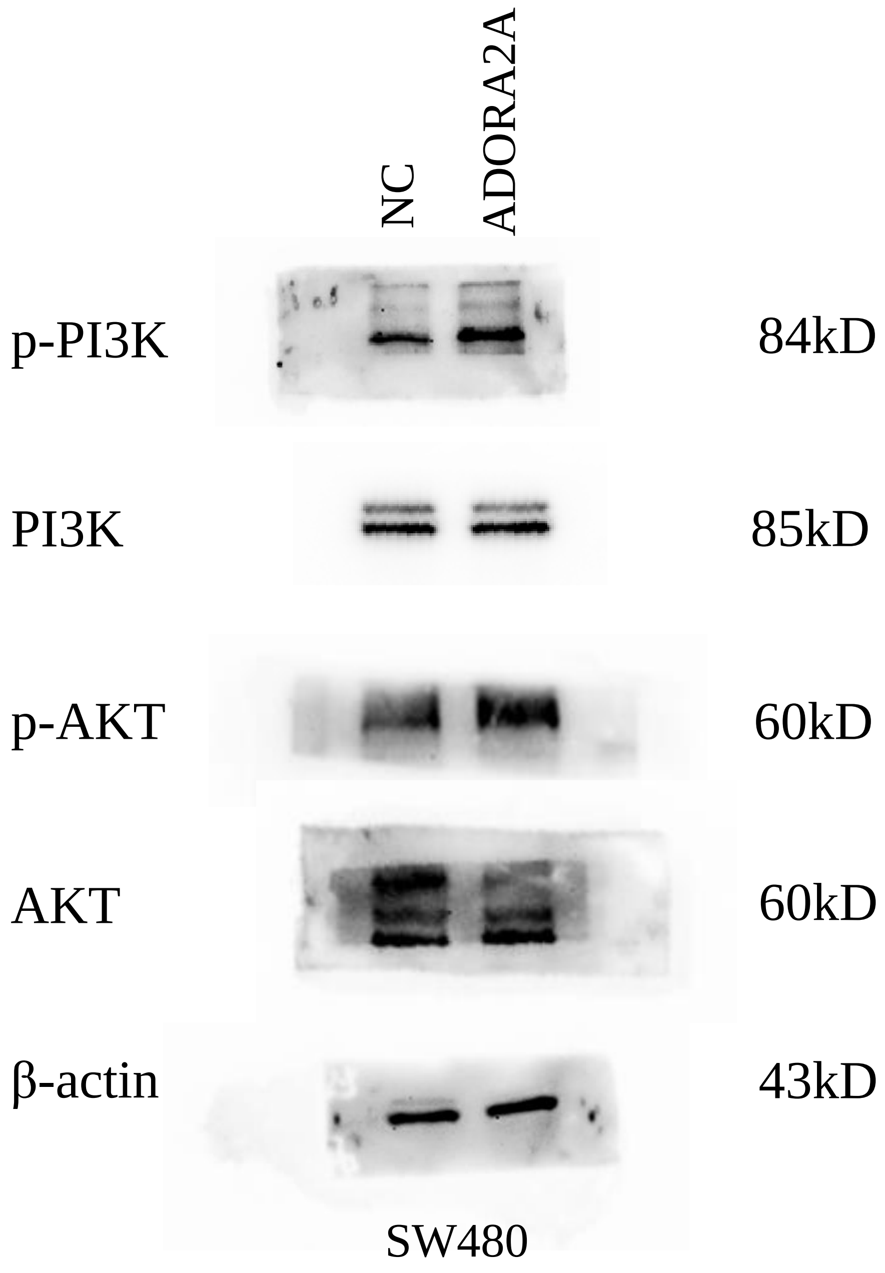

Supplement: Supplementary file 1 — Supplementary Information. [file 41598_2023_46521_MOESM1_ESM.pdf]
